# Detection of Nitroaromatic and Peroxide-Based Explosives with Amine- and Phosphine-Functionalized Diketopyrrolopyrroles

**DOI:** 10.1021/acsami.3c02714

**Published:** 2023-05-31

**Authors:** Monika Warzecha, Graeme Morris, Andrew J. McLean, Jesus Calvo-Castro, Callum J. McHugh

**Affiliations:** † EPSRC CMAC Future Manufacturing Research Hub, c/o Strathclyde Institute of Pharmacy and Biomedical Sciences, Technology and Innovation Centre, 99 George Street, Glasgow G1 1RD, U.K.; ‡ School of Computing, Engineering and Physical Sciences, University of the West of Scotland, Paisley PA1 2BE, U.K.; § School of Life and Medical Sciences, University of Hertfordshire, Hatfield AL10 9AB, U.K.

**Keywords:** diketopyrrolopyrroles, explosives, nitroaromatics, peroxides, fluorescence detection, thin films

## Abstract

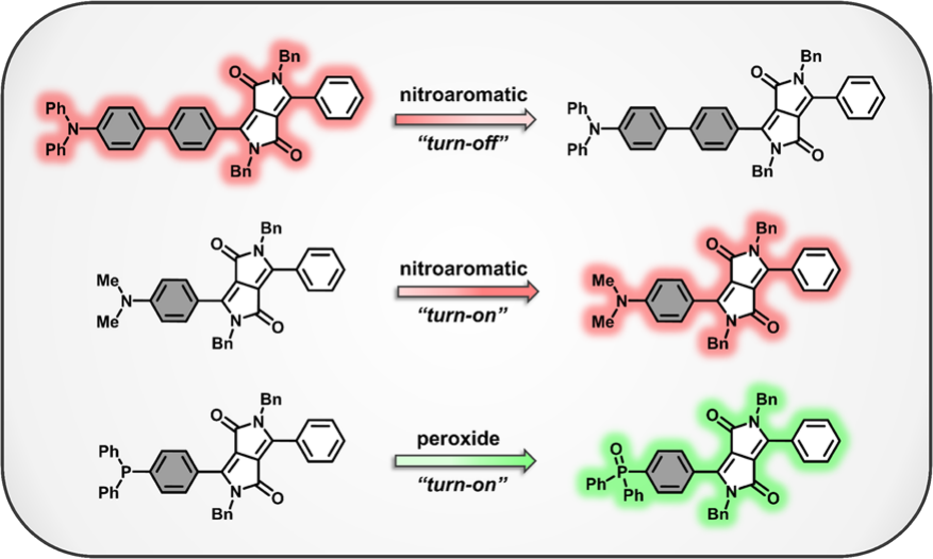

Effective strategies for the detection and identification of explosives are highly desirable. Herein, we illustrate the efficient optoelectronic detection of nitroaromatic and peroxide-based explosives using amine- and phosphine-substituted diketopyrrolopyrroles. Selective quenching and an unprecedented enhancement of thin-film emission in the presence of nitroaromatic vapors are demonstrated *via* the judicious choice of amine substituents. The modulation of fluorescence emission in each case is shown to be dominated by electronic and thermodynamic effects, the vapor pressure of explosives, and the thin-film morphology. For peroxide detection, we describe an approach exploiting redox-mediated functional group transformation. The rapid oxidation of triphenylphosphine to phosphine oxide with hydrogen peroxide affords a significant increase in fluorescence emission, facilitating the sensitive turn-on detection of an important class of explosives at ppb concentrations.

## Introduction

In a world of rapidly evolving global threats and uncertainties, defined by diverse and sophisticated enemy activity and terrorist attacks, innovative approaches to enable the identification of hidden ordnance, improvised explosive devices (IEDs), and landmines command significant importance.^1−3^ Military-grade nitroaromatic explosives, such as 2,4,6-trinitrotoluene (TNT), and volatile impurities, such as 2,4-dinitrotoluene (DNT), endure as key detection challenges, often present in decommissioned landmines, representing both a homeland security issue and environmental concern.^4−12^ These materials, and peroxide-based explosives, such as triacetone triperoxide (TATP) and hexamethylene triperoxide diamine (HMTD), are also widely deployed in IEDs, gaining prominence in recent years because of their emergence as energetic components in a number of high-profile public bombings.^13^ Because of their varied chemical and physical properties, broad-class detection of explosives remains a complex problem. Conventional approaches for the direct detection of explosive residues and vapors rely on instrumental methods such as X-ray spectroscopy, ion mobility spectrometry, mass spectrometry, thermal energy analysis, Raman scattering, and nuclear quadrupole resonance, which are often limited to laboratory environments.^4^ The gold standard for illicit substance detection is the use of canines; however, they are disadvantaged through high costs and certain operational limitations.^14,15^ In recent years, significant progress has been made in optical-based methods, and contemporary reviews highlight that this area shows great potential in the quest for a detection grail.^16,17^ To date, the most successful strategies are those focused on fluorescence quenching or enhancement, with the modulation of the analyte response achieved *via* processes including photo-induced electron transfer (PET), energy transfer, and functional group interconversion. Ubiquitous in the detection of nitroaromatic explosives by fluorescence quenching are conjugated polymers based on poly-(phenylene-ethynylene) and poly-(phenylene-vinylene) derivatives developed by the Swager group.^17,18^ Pentiptycene and dibenzochrysene derivatives commercialized in the Fido X systems are selective and sensitive, with vapor response to nitroaromatic explosives in the femtogram range.^19^ Other notable approaches to the fluorescence quenching detection of nitroaromatics include the use of polysilanes and polymetalloles,^20^ small-molecule microarrays,^21^ porous silica,^22^ nanowire arrays,^23^ nanofibril films,^24^ molecularly imprinted polymers,^25^ calix[4]arenes,^26^ and metal–organic frameworks.^27^ Current methodologies for the detection of peroxide explosives, such as TATP, largely involve nonoptical methods. Fluorescence enhancement strategies coupled with separation techniques and enzymatic treatments have been reported, although they often require complex analyte pretreatments, limiting their effective implementation.^16^ More recently, elegant approaches for the detection of peroxide explosive traces and vapors have been developed, largely focused on the hydrolysis of explosives to hydrogen peroxide and detection of this species,^28,29^ where signal transduction is achieved *via* selective functional group transformation and fluorescence enhancement of nonemissive probe materials.

Diketopyrrolopyrrole (DPP) small molecules and polymers are a world-leading class of π-conjugated organic semiconductors that display highly tuneable and functional optoelectronic properties.^30^ Many of these materials exhibit efficient fluorescence emission and demonstrate robust light and thermal stability. Accordingly, they make promising candidates as signal transducers and molecular probes in optical and electronic sensing environments. Our group has previously reported two simple DPP architectures for the detection of explosives, whose solutions and thin films undergo efficient fluorescence quenching in the presence of nitroaromatics such as TNT, DNT, and nitrobenzene (NB).^31^ In our seminal communication, the vapor response of these materials toward nitroaromatic targets was shown to be strongly influenced by their solid-state structure and thin-film morphology. Rather surprisingly, we can only find two subsequent reports of DPPs being employed in the detection of explosives: an organic field-effect transistor (OFET) approach incorporating a polymeric DPP active layer^32^ and, more recently, a DPP small molecule for the solution-state detection of nitrophenol derivatives by fluorescence quenching; however, notably, this molecular system is not generally classed as a contemporary explosive target.^33^ We have also recently described a highly effective, neutral DPP small molecule for cell imaging based on phosphine chemistry, which displays organelle-specific accumulation within cell mitochondria at nanomolar concentrations.^34^ Importantly, oxidation of the phosphine functional group in this probe was highly effective in precluding PET, resulting in a significant increase in the fluorescence quantum yield of phosphine oxide and superior cell imaging behavior.

Inspired by these observations, in this study, we illustrate the sensitive fluorescence detection of nitroaromatic and peroxide explosives using amine- and phosphine-based DPP chemistries. For vapor-phase nitroaromatic sensing, our molecular design strategy was motivated to enhance the selective modulation of DPP fluorescence emission through the exploitation of well-established and favorable interactions of aromatic nitro compounds with Lewis and Brönsted Lowry bases. Such interactions have been studied for over a century, and a variety of products has been identified, including donor–accepter charge-transfer π-complexes, σ-based Meisenheimer complexes, radical ions by electron transfer, and carbanions by proton abstraction.^35−37^ Herein, we highlight the unique performance of DPP probes containing tertiary aromatic amines, which were anticipated to form selective donor–acceptor complexes upon reaction with TNT, DNT, and NB. In solution, the DPP steady-state fluorescence emission is quenched upon exposure to each of the nitroaromatics under aerated conditions. From fluorescence lifetimes and the Stern–Volmer analysis, the largest bimolecular quenching rate constants were observed from a dimethylamine-functionalized DPP, and the results are consistent with the formation of a ground-state fluorophore–nitroaromatic complex. In the solid state, the modulation of DPP thin-film emission in the presence of nitroaromatic vapors is selectively tuned by the type of DPP amine employed. The exposure of amorphous and seeded films of the dimethylamine derivative to NB and DNT vapors afforded a remarkable enhancement of the thin-film emission. This highly unusual fluorescence turn-on behavior is accompanied by changes in film absorption and emission spectra and morphology, which we propose are consistent with the loss of DPP intramolecular charge-transfer character resulting from ground-state interactions similar to those observed in solution. For peroxide detection, we employed a DPP phosphine, which in solution is rapidly converted to phosphine oxide upon treatment with H_2_O_2_. This redox-mediated functional group transformation is accompanied by a large increase in the fluorescence emission of the DPP probe, providing a novel and sensitive method for the detection of an important explosive metabolite at environmentally relevant concentrations. For both types of explosive classes, such distinctive fluorescence turn-on approaches are highly desirable and represent an emerging frontier in sensing explosives due to their enhanced sensitivity and selectivity.^16^

## Experimental Section

### Synthesis of DPPs (1–9)

Compounds (1)–(9) were prepared and characterized according to the literature methods described by us previously.^31,34,38,39^ Unless otherwise stated, all starting materials and reagents were obtained from Sigma-Aldrich and used as received.

### Optical Spectroscopy and Photophysics

Spectroscopic-grade dichloromethane was purchased from Sigma-Aldrich and used as received. Absorption spectra of solutions and thin films were recorded using a Perkin-Elmer Lambda 40 ultraviolet/visible (UV/vis) spectrophotometer. Unless otherwise stated, thin-film absorption spectra were corrected for scattering. In short, a part of the spectrum was selected, where the absorbance was caused only by scattering. A polynomial was then fitted to this part of the spectrum using a least-squares fit to the logarithm of the absorbance. Using the coefficients determined from the fit, the scatter contributions at all other wavelengths were calculated and then subtracted from the measured spectrum to obtain the absorbance of the film. Reflectance spectra of solid powders were obtained using a Perkin-Elmer Lambda 9 UV/vis spectrometer equipped with a Perkin-Elmer integrating sphere attachment and converted to absorption spectra using the Kubelka–Munk function. Emission spectra were collected in right angle mode for solutions and in front face mode for solids and thin films using a Perkin-Elmer LS50b luminescence spectrometer equipped with a Hamamatsu R928 photomultiplier tube (185–900 nm). Excitation wavelengths for each of the DPPs were coincident with the absorption maxima obtained from their UV–visible absorption spectra. Fluorescence emission spectra were corrected for the photomultiplier response using the manufacturer’s correction method. Fluorescence quantum yields for DPPs (1)–(5) and (9) were experimentally determined following a previously described method,^40^ utilizing an integrating sphere (Labsphere, 6″ inner diameter). Samples were irradiated employing a Xe lamp (λ_exc_ = 469 nm), and the signals were collected by an optical fiber coupled to a Maya2000ProUV-NIR spectrometer. Fluorescence quantum yields for DPPs (6)–(8) were determined by simultaneous multiwavelength thermal lens and photoluminescence spectroscopy.^39^ Lifetime measurements of DPPs 1–9 were carried out by using a time-correlated single-photon-counting instrument (Edinburgh Instruments FLS 920). To excite the samples, a picosecond pulsed EPLED 485 (pulse width, 482.0 nm; FWHM, 100.6 ps) was used. Deconvolution analysis of the decay kinetics yielded the fluorescence lifetimes. A light-scattering Ludox solution was used to obtain the instrument response function.

### Electrochemical Analysis

Electrochemical measurements were performed by cyclic voltammetry using a Metrohm Autolab PGSTAT30 potentiostat. A conventional three-electrode configuration was employed with a glassy carbon working electrode (BAS Inc.), a platinum wire auxiliary electrode (BAS Inc.), and a Ag/AgCl (0.1 M NaCl) reference electrode (BAS Inc.). The glassy carbon electrode and platinum working electrode were electrochemically cleaned before and after each measurement using a 0.5 M sulfuric acid solution by running oxidative scans at 0.05 and 0.1 V s^–1^, respectively. In addition, the surface of the glassy carbon electrode was polished before any measurement utilizing the cleaning kit provided by the manufacturer. Ferrocene was employed as the internal standard reference and tetrabutylammonium hexafluorophosphate (98%, Sigma-Aldrich) as the supportive electrolyte at a concentration of 0.1 M in dichloromethane. The DPP concentrations were set to 10^–3^ M in dichloromethane, and all measurements were conducted under dry argon (BOC) by outgassing the voltammetry cells for 10 min.

### Solution-State Fluorescence Quenching with Nitroaromatics

Steady-state fluorescence quenching experiments were carried out in HPLC-grade dichloromethane. The quenchers, NB (99%) and DNT (97%), were purchased from Acros Organics and Sigma-Aldrich, respectively, and used without further purification. Small quantities of TNT were obtained from the University of Strathclyde and used as received in solution. The Stern–Volmer experiments were performed employing no less than 5 aliquots of each quencher with increasing concentrations while maintaining a constant fluorophore concentration. The solution absorbance was measured before and after quencher addition so that the fluorescence intensity could be corrected for the possibility of variation in the absolute photon absorption, and the presence of ground-state complexes could be investigated at high quencher concentrations. Good linearity and intercepts were obtained for all of the DPP/quencher combinations below a quencher concentration of 0.01 M, and no evidence of an inner-filter effect was observed. From the resulting Stern–Volmer plots, the Stern–Volmer constant, *K*_sv_, was extracted from the slope of the straight line and employed with the fluorophore lifetime, τ_0_, to determine the bimolecular quenching rate constant, *k*_q_. The thermodynamic driving force for electron transfer was estimated by determination of the Gibbs free energy, Δ*G*, which was calculated by eq 1, where *E*°(*D*^+^/*D*), Δ*E*_0,0_, and *E*°(*A*/*A*^–^) are the DPP oxidation potential, DPP singlet excitation energy, and nitroaromatic reduction potential, respectively.
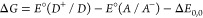
1

### Thin-Film Fabrication and Solid-State Optical Characterization

Thin films of DPPs (6) and (7) were prepared by spin coating onto silica disks (20 mm diameter) from dichloromethane solution using an SPC Spin 150 Coater. Amorphous thin films were prepared by spin coating solutions that had been filtered through a 0.45 μm frit, while more highly ordered films were obtained by spin coating solutions containing microcrystalline seeds of DPP. After spin coating, the thin films were dried at room temperature before analysis or quenching experiments. Films of varying thicknesses were prepared by changing the concentration of the DPP solution used during spin coating. The film thickness was measured using a Veeco Dektak^3^ST surface profiler, and scanning electron microscopy (SEM) images of the films were obtained using a Hitachi S4100 cathode field-emission SEM, employing an Oxford Instruments Germanium EDX detector with a resolution of 115 eV. Optical characterization of thin films by UV–vis absorption spectroscopy and fluorescence spectroscopy was described earlier.

### Solid-State Fluorescence Quenching

Quenching of the thin-film emission was conducted using a modified version of the method reported by Swager,^17,18^ where the UV–vis absorption and emission spectra of the film were recorded before and then during exposure to equilibrated headspace vapors of DNT or NB. To carry out the time dependence analysis, films of DPP 6 and 7 were placed in a prefabricated sample holder designed to fit into the UV–vis and fluorescence instruments described earlier. This ensured that the same part of the film was investigated during quenching to avoid issues related to film inhomogeneity, such as variable photon absorption. For exposure to vapors of the explosive, the sample holder with the film was placed into a separate prefabricated chamber containing a small quantity of the nitroaromatic covered by cotton wool, which had been pre-equilibrated overnight at 25 °C to ensure a saturated headspace of nitroaromatic vapor (180 ppb of DNT and 300 ppm of NB).^18^ The film sample holder was attached to the chamber, presenting the film surface to the nitroaromatic vapor and avoiding any direct contact of the film with the condensed explosive material. During quenching experiments, the sample holder with the film was removed for spectroscopic investigation and replaced carefully into the sample chamber to minimize any possible loss of nitroaromatic headspace vapor.

### Solution-State Fluorescence Enhancement with Peroxides

All solvents were of spectroscopic grade. Dichloromethane (amylene-stabilized) and ethanol were purchased from Sigma-Aldrich, acetonitrile was purchased from Rathburn Chemicals Ltd., and sodium hydroxide solution (0.10 M) was made up by dissolving 0.99960 g of NaOH in 250 mL of ultrahigh-purity (UHP) water produced from a Purelab Flex Elga water purification system. Hydrogen peroxide (>30% w/v) was obtained from Fisher Scientific at a concentration of 8.851 M and used as received. For H_2_O_2_ sensing experiments, nine samples were made up, with increasing concentrations of H_2_O_2_ (0–100 μM) in a 2.0 μM acetonitrile solution of DPP (9). Dilutions were prepared from a stock solution of H_2_O_2_ in UHP water (1 × 10^–3^ M) using a 500 μL SGE Analytical Science syringe. To attribute the change in fluorescence response upon oxidation of DPP (9) solely to the presence of H_2_O_2_, oxygen and water oxidation were ruled out. A 2.1 μM solution of DPP (9) in both acetonitrile and water was stored for 72 h, after which time, there were negligible changes in the absorbance and fluorescence emission spectra.

### Computational Details

Unconstrained geometries of DPPs (6)–(8) were optimized by means of the wB97X-D density functional^41^ at the 6–31G(d) level, as implemented in Spartan ′20 (v.1.1.1) software.^42^ In all cases, optimized geometries were confirmed by IR analysis, characterized by the absence of any imaginary modes, which is consistent with true equilibria minima.^43,44^

## Results and Discussion

### Design and Synthesis

**Figure 1:**
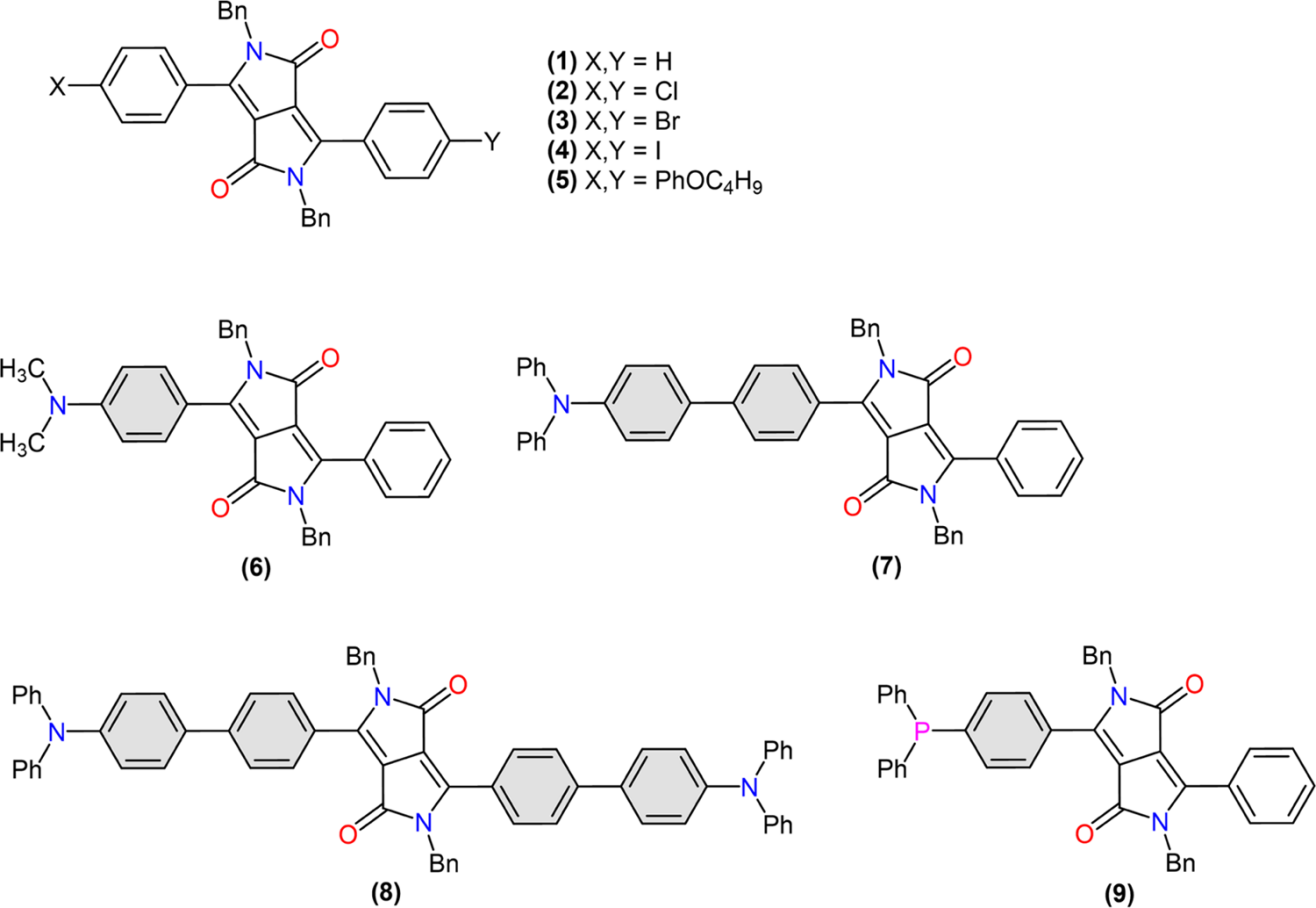
Structures of the DPP compounds (1)–(9) investigated in this work.

The DPP derivatives investigated in this work (Figure 1) were all prepared according to methods reported by us previously. In short, nonsubstituted and halogenated DPPs (1)–(3) were synthesized from the respective aromatic nitrile *via* succinate ester chemistry, followed by benzylation.^31,38^ The iodinated DPP (4), butoxyphenyl derivative (5), and triphenylamine derivative (8) were obtained from (3) *via* halogen exchange and the Suzuki cross-coupling reaction using appropriate boronic acids.^31,38,39^ The dimethylamine derivative (6) was synthesized directly from 4-(dimethylamino)benzonitrile, followed by benzylation.^39^ The triphenylamine derivative (7) was prepared *via* the Suzuki cross-coupling of monobrominated DPP, using 4-(diphenylamino)phenylboronic acid,^39^ while the phosphine (9) was obtained by the palladium-catalyzed phosphination of the respective iodinated derivative with Pd(OAc)_2_ and triphenylphosphine.^34^

### Optical and Electrochemical Characterization in Solution

Steady-state absorption and emission properties and excited-state lifetimes are reported for each of the DPPs in dichloromethane solution (Table 1 and Figure S1). The absorption spectra for each were broad and did not display any vibronic structure. In all cases, the lowest-energy visible bands are attributed to π–π* electronic transitions, consistent with the phenyl-substituted DPPs reported previously.^34^ Fluorescence emission spectra from each of these systems displayed similar spectral shapes, irrespective of the excitation wavelength. In each case, a vibronic progression was observed, with the intensity of the 0–0 band being greater than that of the 0–1 band. Notably, in the halogenated systems (2)–(4), there was no evidence of a heavy atom effect on the fluorescence quantum yield. In the amine derivatives (6)–(8), steady-state absorption spectra were also largely insensitive to the solvent polarity. More significant solvatochromic effects were observed in the emission spectra of the amine derivatives, which were most pronounced in those bearing the triphenylamine substituents (7) and (8).

**Table 1:** Absorption and Fluorescence Emission Maxima (λ_max_^abs^ and λ_max_^em^), Molar Absorption Factor (ε), Photoluminescence Quantum Yield (Φ_f_), Fluorescence Lifetime (τ), and Transition Energy (*E*_0-0_) of Compounds (1)–(9) in Dichloromethane Solution

DPP	λ_max_^abs^ (nm)	ε × 10^4^ (M^–1^cm^–1^)	λ_max_^em^ (nm)	Φ_f_	τ (ns)	*E*_0-0_ (eV)
1	464	1.36	524	0.85	6.39	2.463
2	474	2.03	534	0.83	5.95	2.417
3	475	2.17	537	0.82	5.55	2.408
4	477	2.18	540	0.81	5.54	2.396
5	492	3.49	561	0.81	4.00	2.311
6	516	2.68	576	0.94	4.53	2.248
7	490	2.68	613	0.82	3.76	2.311
8	509	4.27	612	0.85	3.45	2.207
9	476	2.20	539	0.25	5.59	2.419

**Figure 2:**
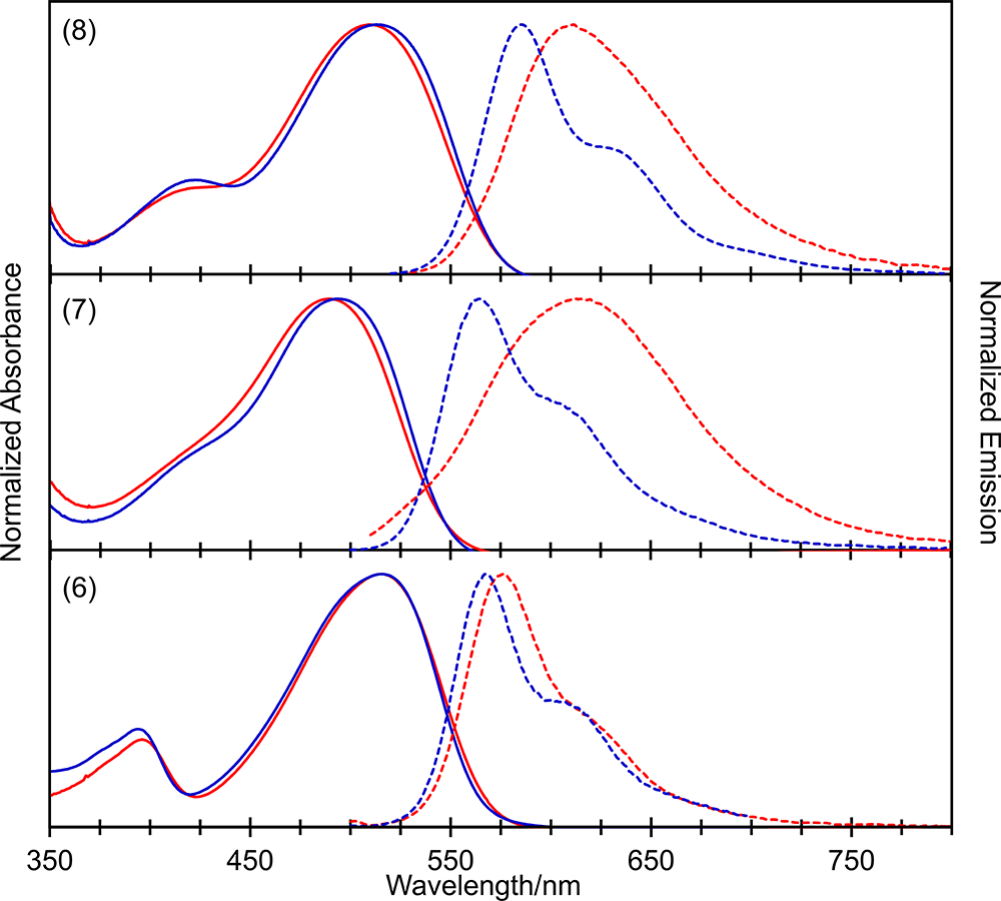
Solvent dependence of steady-state absorbance (solid lines) and fluorescence emission (dashed lines) spectra of amine derivatives (6, bottom; 7, middle; and 8, top) in toluene (blue lines) and dichloromethane (red lines).

In this regard, absorption and emission spectra of (6)–(8) in dichloromethane and toluene solution are shown in Figure 2. We attribute the emission behavior of (7) and (8) to be strongly influenced by the solvent environment and polarity, consistent with fluorescence emission from local excited and/or charge-transfer states. This type of dependence has been previously observed in DPPs containing triphenylamine substituents, where the bathochromic shift and change in emission profile are attributed to the stabilization of twisted intramolecular charge-transfer states in more polar solvent environments.^45,46^ Similarly, in the dimethylamine derivative (6), one would expect solvatochromism to be less pronounced, although still apparent, due to more effective conjugation of the amine group with the core DPP phenyl ring and delocalization of the frontier molecular orbitals (*vide infra*).

**Figure 3:**
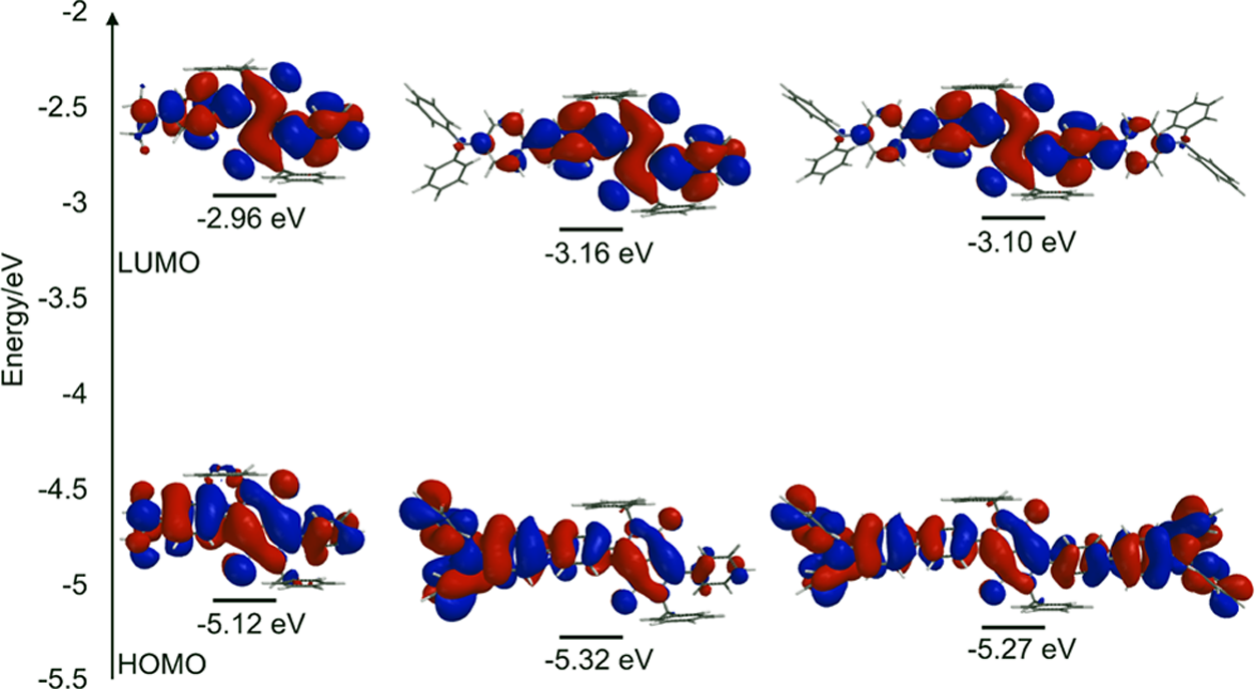
Frontier molecular orbitals for (6) left, (7) middle, and (8) right, computed at the ωB97X-D/6-31G* level. Estimated HOMO and LUMO energies, determined from experimental oxidation and reduction potentials in dichloromethane solution.

Electrochemical characterization of DPPs (1)–(8) was carried out by cyclic voltammetry (CV), and the results are summarized in Figure S2 and Table S1. CV measurements were carried out using a three-electrode system, with a glassy carbon working electrode, a platinum wire counter electrode, and Ag/AgCl as the reference electrode. These data were further used to determine the thermodynamic driving force for electron transfer by estimating the Gibbs free energy for quenching Δ*G* with each of the nitroaromatics in dichloromethane (*vide infra*) and to estimate frontier molecular orbital energies of (6)–(8) from the oxidation onset of ferrocene.^47−50^ The amine derivatives (6)–(8) displayed oxidation potential, *E*_ox_, values of 0.836, 1.033, and 0.989 V and reduction potential, *E*_red_, values of −1.319, −1.157, and −1.185 V in dichloromethane, respectively. Simultaneously, the geometries of these three architectures were optimized by means of density functional theory calculations at the wB97X-D/6-31G* level, and their frontier molecular orbitals were determined (Figure 3). We observe that the highest occupied molecular orbital (HOMO) densities are delocalized throughout the conjugated backbone in each system, expanding into the terminal amine-containing substituents. In turn, it is noteworthy that as the size of the amine substituents increases on progression from (6) to (7) and (8), there is a greater localization of the lowest unoccupied molecular orbital (LUMO) density within the central bislactam core motif, consistent with the solvatochromism observed in these molecules (*vide supra*) as a result of intramolecular excited-state charge transfer.

### Solution-State Fluorescence Quenching with Nitroaromatics

**Figure 4:**
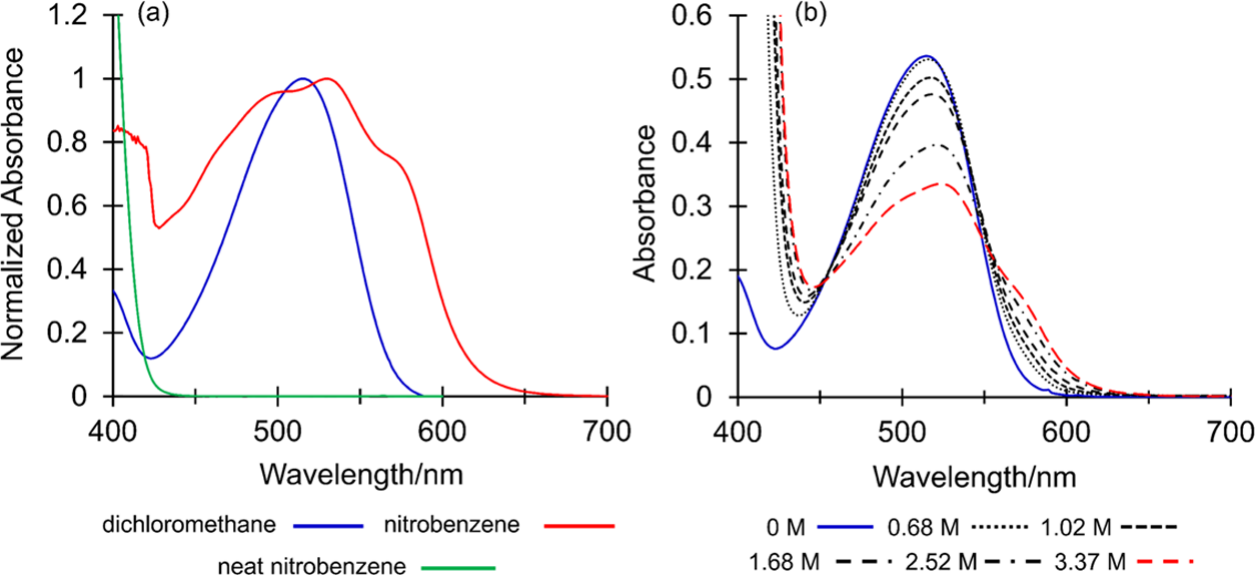
(a) UV–visible absorption spectra of the dimethylamine derivative (6) in dichloromethane (blue line), NB (red line), and neat NB (green line). (b) Titration of (6) in dichloromethane solution (1 × 10^–5^ M) with increasing concentrations of NB, from 0 M (blue solid line) to 3.37 M (red dashed line).

We first evaluated the ability of DPPs (1)–(8) to detect nitroaromatic-based explosives in the solution phase. In all cases, steady-state fluorescence emission in dichloromethane solution was quenched upon exposure to NB, DNT, and TNT under aerated conditions. From fluorescence lifetimes and the Stern–Volmer analysis, the bimolecular rate constants for quenching, *k*_q_, were determined and found to approach diffusion control rates (Table S1).^31^ The Stern–Volmer plots were strictly linear at quencher concentrations below 1 × 10^–2^ M for all of the DPPs; however, at higher quencher concentrations, a positive curvature was observed in the Stern–Volmer plots of the dimethylamine derivative (6) (Figure S3). To probe the possibility of a ground-state complex with this amine, a solution of (6) in dichloromethane was titrated with increasing concentrations of NB, and the UV–visible absorption spectrum was recorded (Figure 4). At increasing concentrations of the nitroaromatic, the λ_max_ of (6) shifted from 516 to 527 nm, and two new bands emerged. The first is a shoulder at approx. 491 nm and the second is a red-shifted stronger band at 579 nm. These changes mirror the UV–visible absorption spectrum of (6) dissolved in pure NB (Figure 4), where the band position and intensity changes are consistent with those reported previously in the characterization of charge-transfer complexes between tertiary aromatic amines and nitroaromatics.^35^

**Figure 5:**
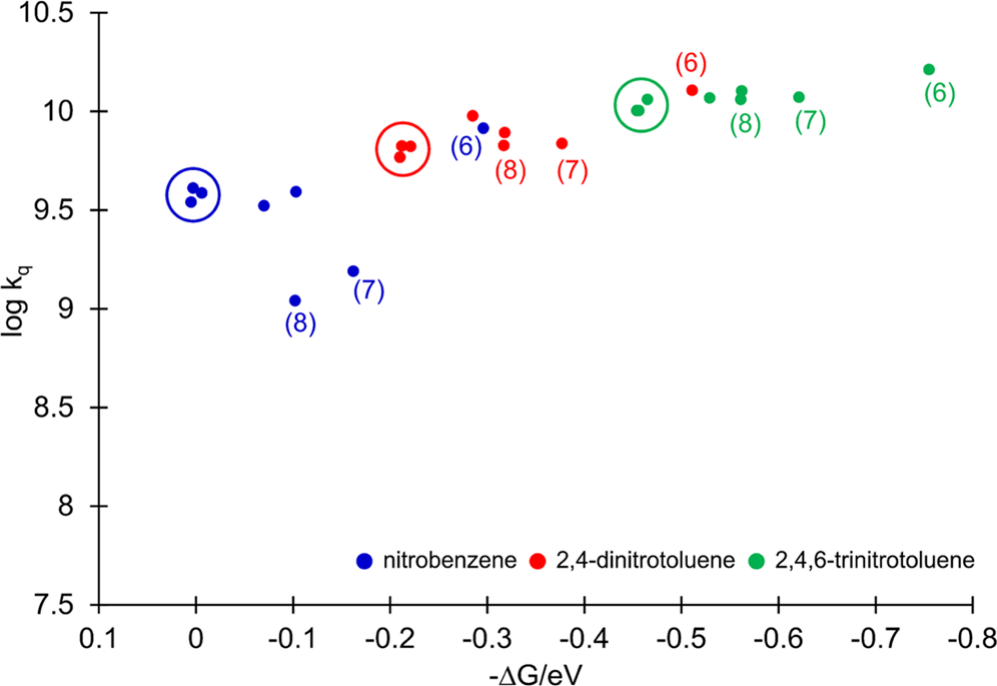
Bimolecular rate constants, *k*_q_, *vs* free energy of reaction, −Δ*G*, for oxidative quenching with NB (blue), DNT (red), and TNT (green) in dichloromethane. Halogenated DPPs (2–4) are circled, and amine-substituted DPPs (6–8) are highlighted in parentheses.

The Gibbs free energy for quenching, Δ*G*, was estimated from the Rehm–Weller equation (eq 1) using DPP and nitroaromatic oxidation and reduction potentials, respectively, and the DPP *E*_0-0_ energies presented in Tables 1 and S1. Based on these data, it can be concluded that electron transfer from the DPP excited state to nitroaromatics should be thermodynamically favorable in all cases (Table S1). The correlation between the bimolecular quenching rate constant in dichloromethane and the free energy of reaction for each DPP/nitroaromatic pairing is presented in Figure 5, as a plot of log(*k*_q_) *vs* −Δ*G*.

In the more exergonic region, data points begin to plateau and approach diffusion control, becoming asymptotic at ca. *k*_q_ = 1.63 × 10^10^ M^–1^ s^–1^, with no evidence of a Marcus inverted region. As the reaction becomes more endothermic, the bimolecular quenching rate constants become lower, as expected. For each nitroaromatic quencher, the halogenated DPPs (2–4) behaved similarly, and little influence of the halide substituent was observed on their *k*_q_ values. In each case, the halide DPPs exhibited a lower thermodynamic driving force but a rate constant similar to those of phenyl (1) and butoxyphenyl (5) derivatives, with *k*_q_ = 3.92 × 10^9^, 7.81 × 10^9^, and 1.27 × 10^10^ for (1) and 3.33 × 10^9^, 9.50 × 10^9^, and 1.17 × 10^10^ M^–1^ s^–1^ for (5) with NB, DNT, and TNT, respectively.

For the triphenylamine-substituted DPPs, a noticeably lower rate constant was observed with NB when compared to the dimethylamine derivative (6) (*k*_q_ = 8.22 × 10^9^, 1.55 × 10^9^, and 1.10 × 10^9^ M^–1^ s^–1^ for (6), (7), and (8), respectively) despite their comparable thermodynamic driving forces. As the endothermicity of the reaction increases, coupling between the molecules of the reactants ought to be maximized for effective electron transfer, and so closer contact is required. Within the inner-sphere formalism for electron transfer, the reorientation time of the reactants must be considered, representing the rate-determining step.^51−53^ In oxidative quenching, electron transfer occurs from the excited state of the fluorophore to the quencher. The former is often approximated as the ground-state LUMO orbital. Thus, such processes can be evaluated from the participating frontier molecular orbitals, which are the LUMO orbitals of the fluorophore and quencher. The LUMO orbitals of triphenylamines (7) and (8) are shown in Figure 3. Much of the electron density in these systems is distributed throughout the DPP bislactam core, and for each of the nitroaromatic quenchers, the LUMO electron density is largely delocalized throughout their conjugated core.^23^ Given the requirement for close contact between the fluorophore and the quencher to maximize electronic coupling, steric effects, as in the case of (7) and (8), both of which contain bulky and sterically demanding triphenylamine groups, are anticipated to play a pivotal role. The quenching rate constant for the dimethylamine derivative (6) and NB was approximately twice that observed for DPPs (1)–(5) and 6 times higher than those of (7) and (8), with *k*_q_ = 8.22 ×10^9^, 1.55 ×10^9^, and 1.10 ×10^9^ M^–1^ s^–1^ for (6), (7), and (8), respectively. This is consistent with a more exergonic driving force Δ*G* (−0.296, −0.162, and −0.102 eV for (6), (7), and (8), respectively) for the dimethylamine derivative, a more accessible DPP LUMO orbital, and the existence of ground-state charge-transfer interactions between this fluorophore–quencher pair.

In systems exhibiting larger driving forces, close contact is not a prerequisite for efficient electron transfer, which is primarily controlled *via* orientational nonspecific interactions between reactant molecules and their outer-sphere electron-transfer rate constants denoted by a semi-classical Marcus dependence on free energy.^54−56^ For the halogenated (2–4), phenyl (1), and butoxyphenyl (5) DPPs, the quenching mechanism with DNT and TNT was similar to that of NB, where the more exergonic driving force with these nitroaromatics afforded higher values of *k*_q_. For triphenylamines (7) and (8), the quenching rate constants (*k*_q_ = 6.88 × 10^9^ and 1.18 × 10^10^ M^–1^s^–1^ for (7) and 6.72 × 10^9^ and 1.15 × 10^10^ M^–1^ s^–1^ for (8) with DNT and TNT, respectively) were comparable to those observed for DPPs (1)–(5) and, despite their larger driving forces, consistent with a larger total reorganization energy, which might be anticipated, based on the size and conformational flexibility of the triphenylamine substituents in these systems. For the dimethylamine derivative (6), *k*_q_ increased according to the driving force with both DNT and TNT. In contrast to quenching with NB, however, *k*_q_ was only ca. 50% higher than that observed for the other DPPs (*k*_q_ = 1.28 × 10^10^ and 1.63 × 10^10^ M^–1^s^–1^ for DNT and TNT, respectively), consistent at this low concentration to a plateau of *k*_q_ arising from diffusion-limited control.^31^

### Thin-Film Fabrication and Solid-State Optical Characterization

**Figure 6:**
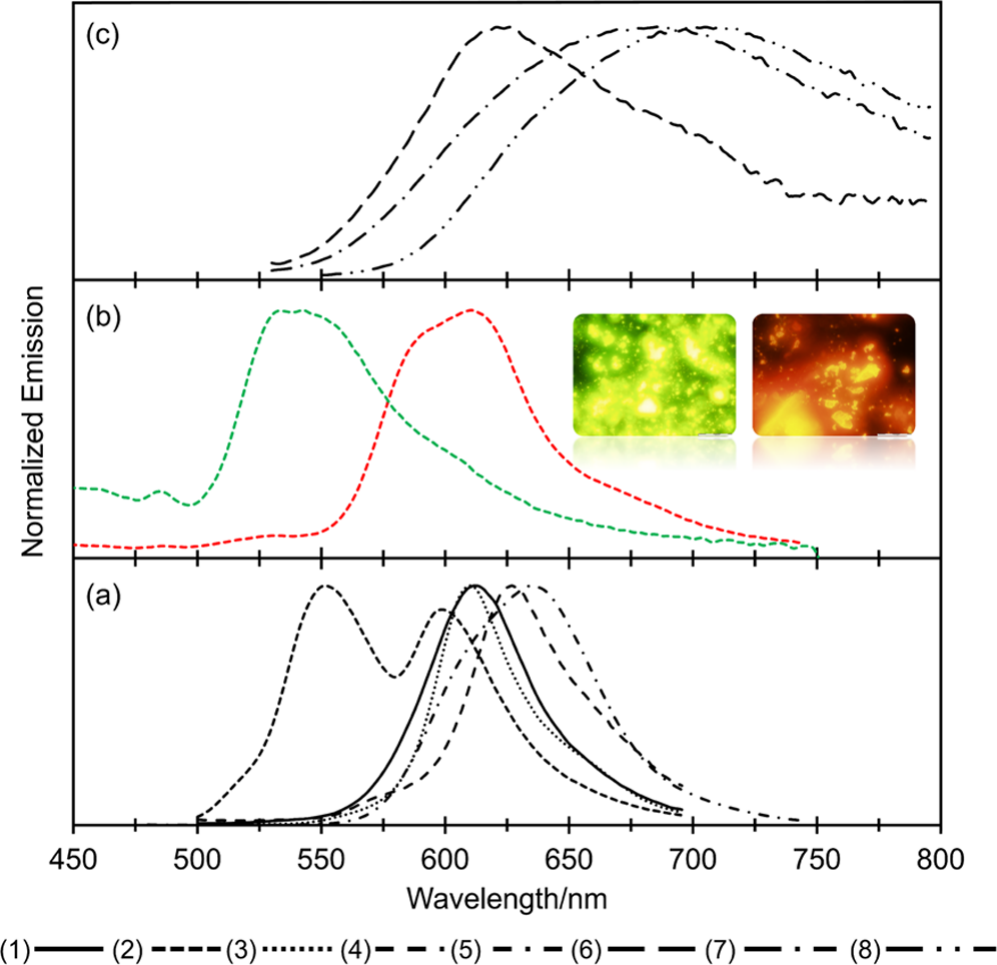
Solid-state fluorescence emission spectra of (a) DPPs (1)–(5), (b) α and β phases (green and red lines, respectively) of DPP (2), with the insets showing fluorescence microscopy images of the α-phase (left) and β-phase (right) single crystals, and (c) DPPs (6)–(8).

Inspired by the solution-phase quenching performance of DPPs (1)–(8), we next proceeded to investigate their solid-state behaviors. The Kubelka–Munk-derived absorption spectra from polycrystalline powders of DPPs (1)–(5) were broad and hypsochromically shifted compared with those in solution (Figure S4). This is consistent with the blue shift expected from slipped π-stacking interactions, which have previously been reported in these systems.^38^ For the polymorphic DPP (2), absorption spectra of the known crystal phases (λ_max_ = 477 and 521 nm for the α and β phases, respectively) are consistent with their solid-state conformations, where the β-phase is considerably more planar in structure (i.e., the torsion of the core phenyl ring is lower with respect to the plane of the DPP core) and hence the bathochromic shift observed with respect to the α-phase.^38^ Solid-state absorption spectra from powders of amines (6)–(8) were similar to the band shape obtained in solution; however, a bathochromic shift was observed in all cases (λ_max_ = 516/531, 490/501, and 509/521 nm for (6), (7), and (8), respectively, in dichloromethane solution/powder). Solid-state fluorescence emission spectra from all of the powders displayed significant bathochromic shifts compared to their dichloromethane solutions (Figure 6). Of particular note are the dramatic differences in the emission maxima from the two crystal phases of DPP (2) (541 and 612 nm for the α and β phases, respectively), again consistent with their distinct crystallographic packing and molecular conformation. Emission spectra of derivatives containing amines (6)–(8) were considerably red shifted. However, aided by the thin-film data (*vide infra*), we attribute the large Stoke′s shift in the powder form to self-absorption.

**Figure 7:**
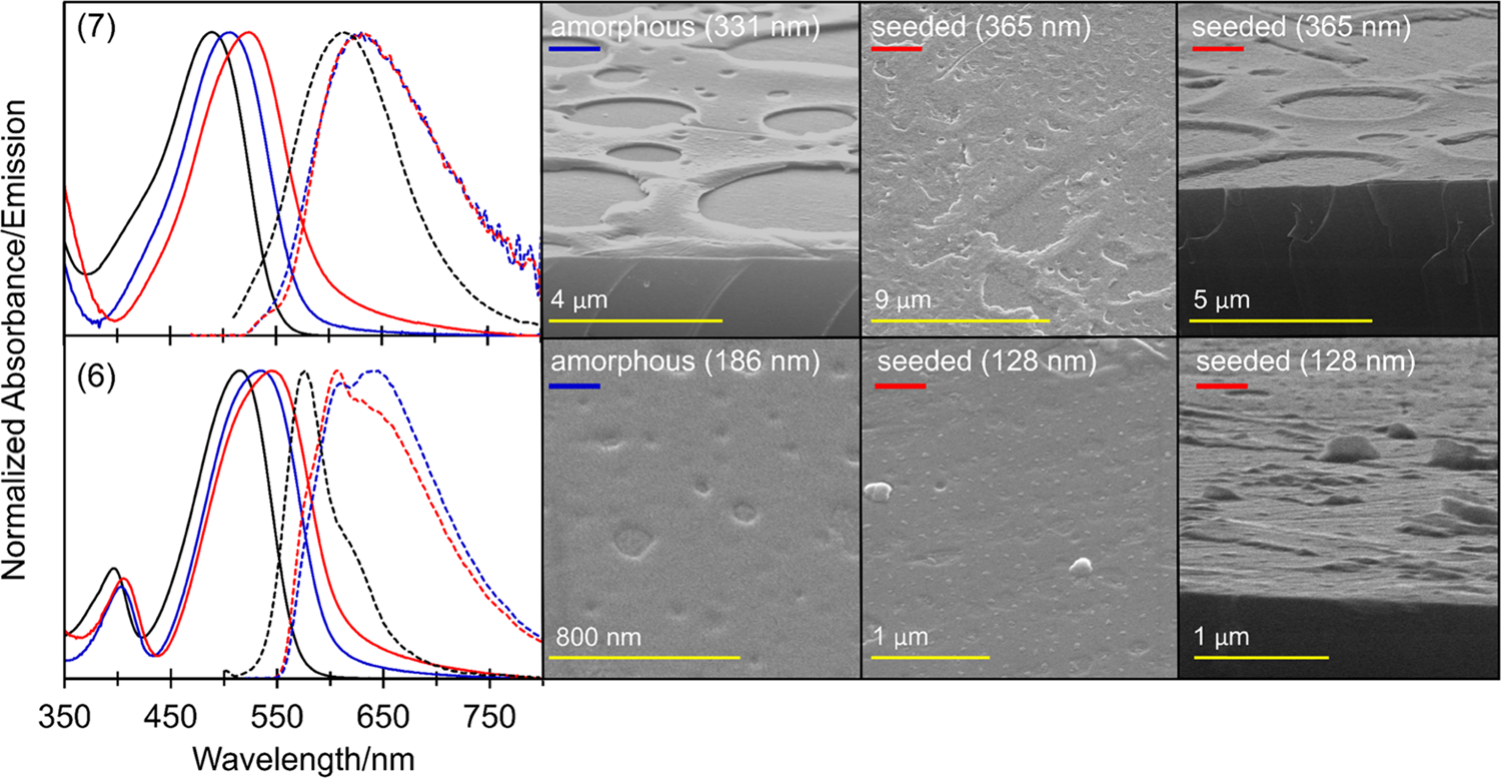
Absorption (solid lines) and emission (dashed lines) spectra from solution (black), amorphous (blue), and seeded (red) thin films of (6) and (7). SEM images of amorphous and seeded thin films of (6) (bottom) and (7) (top).

We have previously demonstrated that fluorescent thin films of DPPs displaying varying thicknesses, structures, and morphologies can be prepared on SiO_2_ by spin coating (1) and (5) from dichloromethane solution.^31^ In this report, we focus our attention on the fabrication of films prepared from amines (6) and (7). Films obtained from triphenylamine derivative (8) were not investigated due to the lower solubility of this material. Amorphous films of (6) and (7) were prepared from filtered dye solutions, while films exhibiting higher long-range structural order were obtained *via* seeding during the spin-coating process. Film thickness and morphology were determined by surface profiling and SEM analysis, with the optical behavior characterized by steady-state absorption and emission spectra. Electronic spectra and SEM images of amorphous and seeded films of varying thicknesses from (6) and (7) are shown in Figure 7. Absorption spectra of both types of films for (6) and (7) were similar to those observed in dichloromethane solution (λ_max_ = 516/534/544 and 490/507/524 nm for (6) and (7), respectively, for solution, amorphous, and seeded films) consistent with a red shift associated with enhanced order in the films. In all cases, the absorption bands were broad and did not exhibit a vibronic structure. Thin-film emission spectra for (7) were similar to those in dichloromethane solution, displaying no vibronic structure. The emission envelopes of these films were slightly red shifted, with the maxima (λ_max_ = 613, 631, and 633 nm for solution, amorphous, and seeded films, respectively) indicative of a similar emitting species in each case. The emergence of a red-shifted tail in both films prepared from (7) may be attributed to charge-transfer emission, consistent with its solvatochromic behavior and observed in the films as a result of favorable solid-state-mediated molecular conformations. Film emission spectra for (6) displayed a more significant red shift compared to solution, with considerable broadening and a more pronounced vibronic structure, with maxima (λ_max_ = 576, 612/645, and 607/642 nm for solution, amorphous, and seeded films, respectively) typical of enhanced structural order in these films. The ratio of the 0–0 and 0–1 band intensities in the emission spectra suggest the presence of H and J-aggregations in the amorphous and seeded films of (6), respectively.^57^ A similar behavior has been identified by us previously in films prepared from DPP (5), consistent with polymorphism in *N*,*N*′-dibenzyl DPPs, and the propensity in these systems toward various slipped co-facial orientations over their long molecular axis.^31,38^ Excitation energies for all of the films were estimated from the crossing points of their absorption and emission spectra and were lower than those observed in dichloromethane solution (*E*_0–0_ = 2.150/2.143/2.248 and 2.204/2.154/2.311 eV for (6) and (7), respectively, from amorphous/seeded films/dichloromethane solutions). From the SEM analysis, amorphous and seeded films from (7) displayed a similar morphology, consisting of a bubble-like structure resulting from solvent evaporation. The texture of the seeded film from (7) was rougher, with a higher surface area, which we attribute to the presence of microcrystalline seed particles during spin coating. Amorphous films of (6) exhibited a more uniform morphology than those prepared from (7), while enhanced structural order in the seeded films of (6) was confirmed by SEM analysis, with supramolecular islands of the dye distributed throughout the film surface.

### Solid-State Fluorescence Quenching with Nitroaromatic Vapor

**Figure 8:**
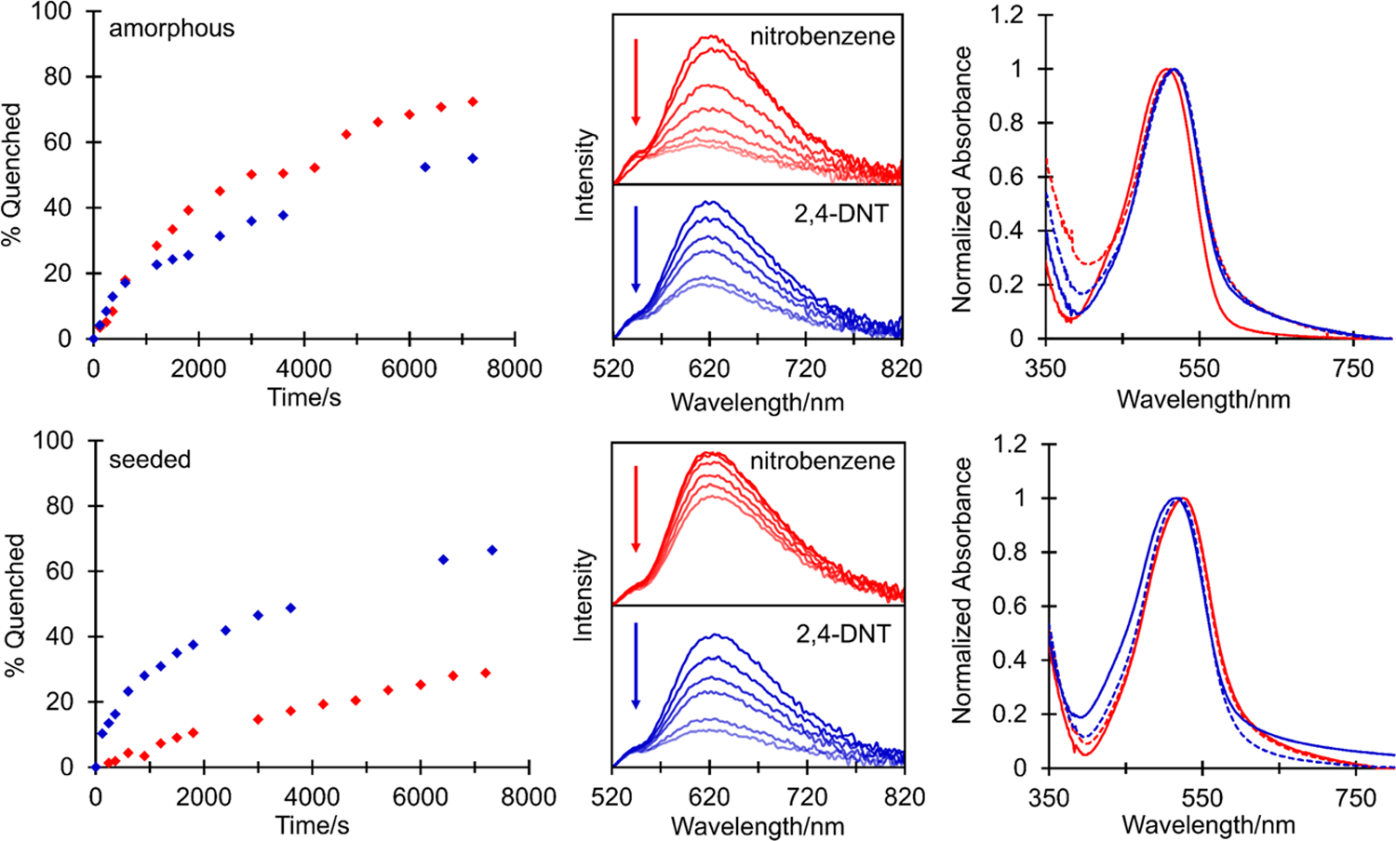
%Fluorescence emission quenched as a function of time (left) for amorphous (top) and seeded (bottom) films of (7) toward NB (red) and DNT (blue) (λ_exc_ = 470 nm). Fluorescence emission spectra (middle) during exposure to NB (red) and DNT (blue) and absorption spectra (right) of amorphous and seeded films of (7) before (solid line) and after (dashed line) exposure to NB (red) and DNT (blue).

Quenching of thin-film emission was investigated as described by us previously, using a modified version of the method reported by Swager for the detection of equilibrated headspace vapors.^17,18,31^ The headspace concentrations of NB and DNT (a headspace marker for TNT)^58^ were determined from their respective vapor pressures at 25 °C (180 ppb and 300 ppm, respectively)^18^ and represent an upper limit of detection for both. We have previously demonstrated for DPP thin films that there is a strong correlation between the film thickness and nitroaromatic quenching dynamics, with a reduction in the response time observed as the film thickness decreases.^31^ In this instance, the film thicknesses employed reflect a balance between the ease of processing, film stability, and overall sensing performance. The fluorescence responses from amorphous and seeded films of (7) to NB and DNT vapors are shown in Figure 8. No significant changes in the band shape or position of the absorption or emission spectra were observed, consistent with a stable film structure and the limited ground-state charge transfer between fluorophore and nitroaromatic observed in solution. Exposure of 206 nm thick amorphous films of (7) to NB vapor resulted in a 40% reduction in emission after 2000 s, reaching 72% at saturation after 7200 s. Treatment of amorphous films (262 nm) with DNT vapor revealed a lower overall quenching response, with 26 and 55% decreases in the emission intensity after 2000 and 7200 s, respectively.

Remarkably, the initial film response to DNT was over 50% faster than that for NB for the first 360 s of quenching (13 *versus* 8% decrease) despite its significantly lower vapor pressure.^18^ This is consistent with the higher reduction potential and higher electron-transfer rate constant for DNT, which over time is superseded *via* the more rapid permeation of NB throughout the amorphous film. The exposure of seeded films (300 nm) of (7) to NB vapor revealed a markedly different behavior, with only 11 and 30% reductions in emission intensities after 2000 and 7200 s, respectively. In contrast, the response of comparable 320 nm thick seeded films to DNT vapor was superior to those of their amorphous equivalents, with 38 and 66% reductions in emission intensities after 2000 and 7200 s, respectively. Notably, the initial response of seeded films to DNT was faster than for amorphous films, with a 35% increase in quenching observed (23 *versus* 17% decrease, respectively) during the first 600 s of exposure. This is in contrast to the 4% reduction in emission intensity over the same time frame with NB. The selective behavior of seeded films toward DNT is striking, especially given the lower vapor pressure of DNT and less negative Δ*G* for electron transfer compared with those of amorphous films, which were characterized by a higher transition energy (*E*_0–0_ = 2.154 and 2.204 eV for seeded and amorphous films, respectively). The poor response from seeded films of (7) to NB is comparable to solution-based quenching and reflects the higher rate constants observed from DNT and TNT. Accordingly, in the more structured seeded environment, we propose a smaller number of accessible fluorophore conformations able to interact with NB, from which electron transfer can occur between DPP and NB LUMO orbitals. This is not such a serious issue in less ordered amorphous films, where the larger driving force and high quencher vapor pressure ensure rapid NB diffusion and more effective quenching. With DNT and seeded films of (7), the larger quencher reduction potential and concomitant driving force ensure that such specific intermolecular interactions are not necessary, while the improved response to DNT compared with amorphous films is consistent with a higher overall surface area in the seeded environment.^31^

**Figure 9:**
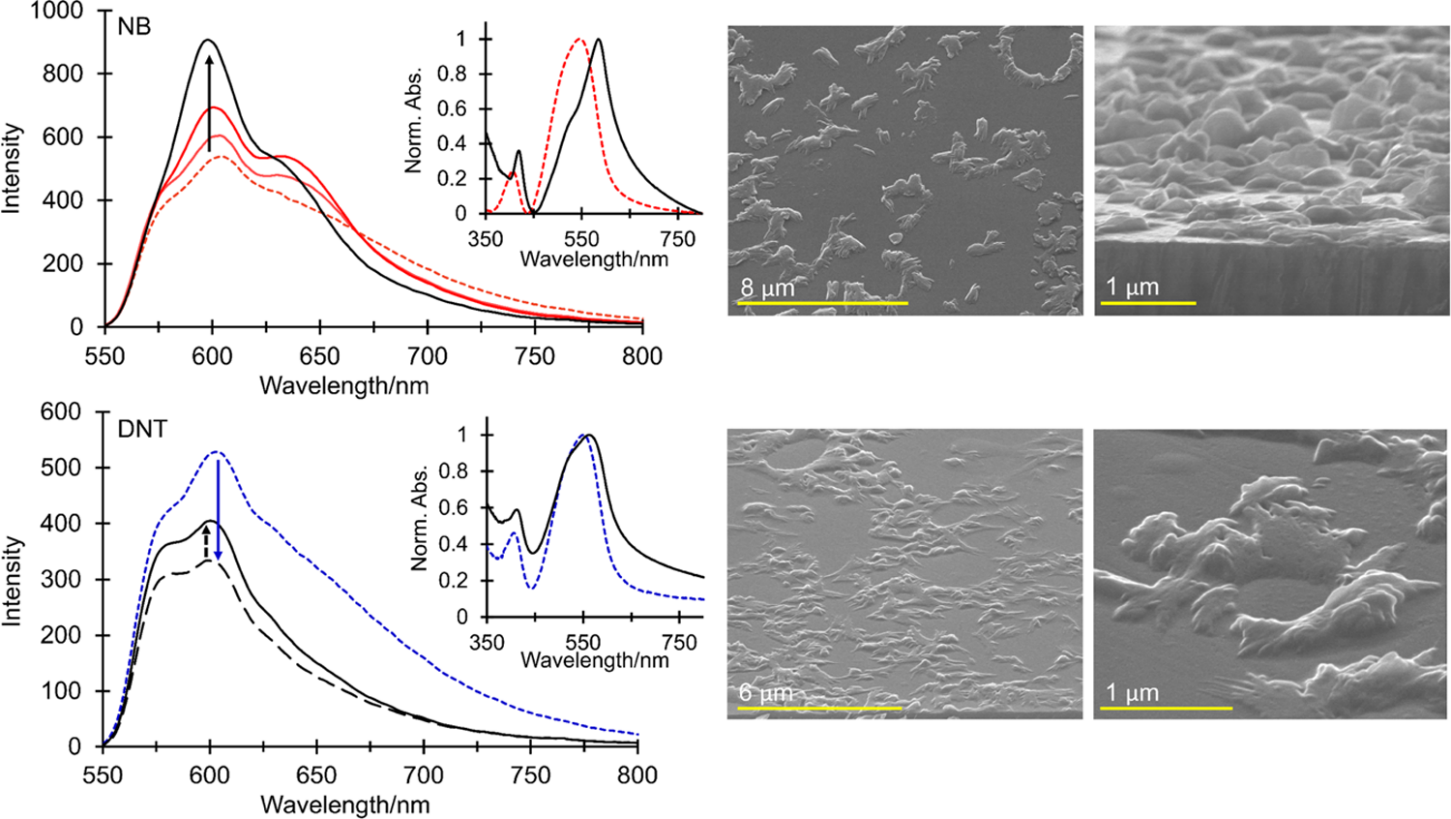
Fluorescence emission response and associated changes in the absorption spectra and morphology of seeded films of (6) to NB (top) and DNT (bottom) vapor (λ_exc_ = 500 nm). In the emission and absorption spectra with NB, the dashed red and solid black lines show pretreatment and saturation, respectively, while the solid red lines in the emission spectra are obtained after 60 and 7200 s exposure. For DNT, the blue line (small dashes) shows pretreatment, the gray line (large dashes) is obtained after 6000 s exposure, and the solid black line at saturation. SEM images show the formation of surface crystallites after exposure to NB (top) and DNT (bottom) at saturation.

The fluorescence response from seeded films of (6) to vapors of NB and DNT is shown in Figure 9. Remarkably, enhanced fluorescence emission was observed in both cases. This effect is unique, with direct emission enhancement in nitroaromatic sensing only being reported in limited cases involving picric acid detection^59−61^ and broad-class nitroaromatic detection achieved only recently *via* a displacement assay.^62^ Exposure of 128 nm thick seeded films to NB vapor afforded a dramatic amplification in the fluorescence intensity with a 30% increase after 7200 s (13% after 60 s), reaching 69% at saturation. The increased emission from these films was accompanied by a slight hypsochromic shift in the λ_max_ of their 0–0 band (604–599 nm) and a reduction in the intensity of longer wavelength charge-transfer transitions, resulting in an overall narrowing of the emission envelope. In turn, the exposure of seeded films (80 nm) to DNT vapor resulted in an initial 37% reduction in emission. However, after approx. 6000 s, quenching was reversed, with a 21% recovery in fluorescence emission at saturation. As observed with NB treatment, the enhanced fluorescence following extended exposure to DNT was accompanied by changes to the emission band shape and position, with a slight hypsochromic shift in the 0–0 band and a narrowing of the emission envelope following the loss of charge-transfer character. Treatment with NB and DNT vapor was further characterized by a significant change in the film morphology and the emergence of extensive surface crystallites, as shown in Figure 9. A shift in the λ_max_ of the film absorption spectra from 554 to 589 nm was also observed, along with the appearance of a shoulder at approx. 524 nm (this change was more dramatic in films treated with NB). The emergence and position of these bands are consistent with those attributed to the formation of a ground-state charge-transfer complex in solutions of (6) and NB (527 and 579 nm) and imply the formation of a solid-state species in the film responsible for the enhanced fluorescence.

Loss of charge-transfer states in the film emission supports this type of interaction and is consistent with a reduction of intramolecular charge separation in the dye, occurring through competitive ground-state interactions between the HOMO of (6) and nitroaromatic LUMO orbitals. Rapid enhancement of fluorescence emission with NB reflects fast complex formation, supported by the high presaturation concentration of NB and the surface area of the film. Initial quenching with DNT is consistent with slower reaction kinetics and is controlled by the lower vapor pressure of DNT, its slower diffusion through the film, and a larger driving force for electron transfer, which are dominant before complex formation and the associated increase in fluorescence. Modulated emission from solvent annealing effects, as described by us previously, were ruled out.^31^ In those examples, changes to the film structure were strictly accompanied by a decrease in fluorescence emission and only minor variations to the relative intensity of the absorption/emission vibronic progression after treatment with NB. Neither annealing effects nor changes to film spectra were observed from exposure to DNT vapor, consistent with its significantly lower vapor pressure.

**Figure 10:**
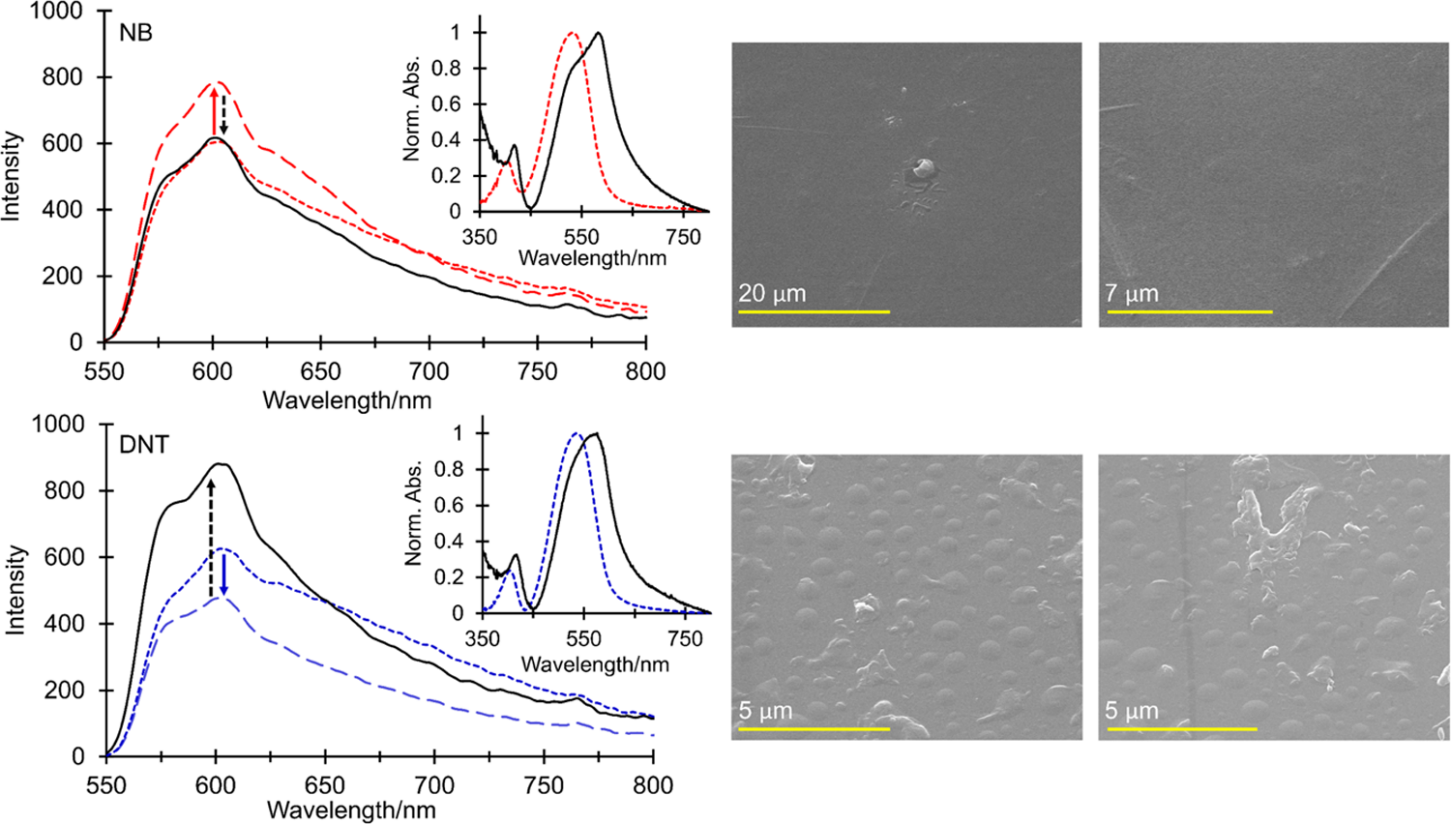
Fluorescence response and associated changes in the absorption spectra and morphology of amorphous films of (6) to NB (top) and DNT (bottom) vapor (λ_exc_ = 500 nm). In the emission and absorption spectra with NB, the small red dashes, large red dashes, and solid black lines show pretreatment, after 600 s, and at saturation, respectively. For DNT, the small blue dashes, large blue dashes, and solid black lines show pretreatment, after 5100 s, and at saturation, respectively. SEM images show a change in morphology and the formation of surface crystallites after exposure to DNT (bottom) but no significant changes in the film morphology after exposure to NB (top) at saturation.

The fluorescence response from amorphous films of (6) to vapors of NB and DNT is shown in Figure 10. Exposure of 63 nm thick films to NB vapor again resulted in the rapid amplification of fluorescence (30% increase after 600 s), which in this case slowly reduced to the starting intensity toward saturation. With DNT vapor, an initial reduction in emission (23% after 5100 s) was observed from 74 nm thick amorphous films, which was again reversed to yield 85% recovery at saturation. No significant changes were observed in the emission envelope for either; however, changes in the absorption spectra were similar to those of seeded films, which we propose are consistent with a bimolecular interaction in the ground state. The rapid initial enhancement of fluorescence with NB was again congruent with a high presaturation concentration of NB and fast complex formation. The following small reduction in fluorescence implies saturation of accessible fluorophore conformations in the amorphous film capable of complex formation, consistent with its less ordered structure and lower overall surface area, leading to a solution-like behavior and quenching of the remaining emissive sites. With DNT, slow complex formation and an initial reduction in fluorescence were again governed by the lower vapor pressure of DNT, its slower diffusion through the film, and the larger Δ*G* for electron transfer with the amorphous film, relaxing the requirement for close contact between the fluorophore and the quencher. Upon complex formation, a large increase in the fluorescence emission is observed until saturation, consistent with a more favorable solid-state interaction between DNT and the amorphous film environment. SEM analysis of the amorphous film morphology at saturation further reflects preferable complex formation with DNT in the solid state, where, in contrast to treatment with NB vapor, the emergence of island structures were observed, similar in appearance to those obtained within seeded film environments (Figure 10).

### Solution-State Fluorescence Enhancement with Peroxides

**Figure 11:**
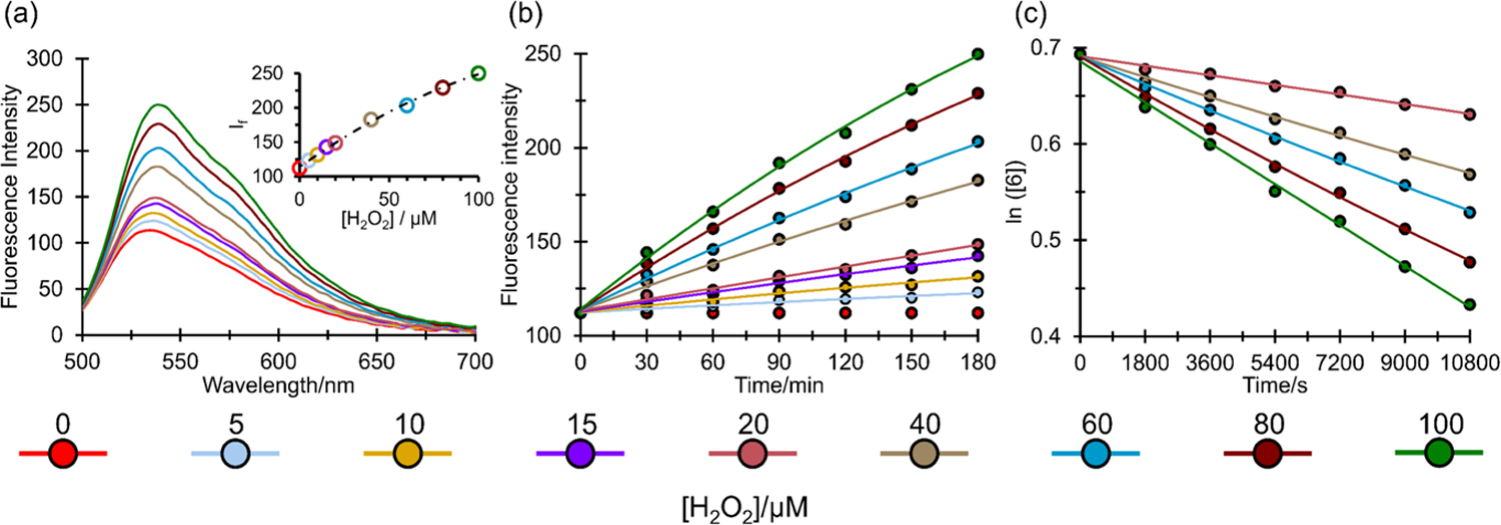
(a) Fluorescence emission spectra of (9) for increasing concentrations of H_2_O_2_ in acetonitrile at *t* = 180 min (inset depicts the fluorescence intensity of (9) at λ_em_ = 539 nm as a function of H_2_O_2_ concentration). (b) Time-dependent kinetic measurement of the fluorescence response of (9) to increasing concentrations of H_2_O_2_ in acetonitrile (λ_em_ = 539 nm; λ_exc_ = 470 nm). (c) Pseudo-first-order linear fits for the oxidation of (9) in the presence of 20–100 μM concentrations of H_2_O_2_ in acetonitrile.

In the remainder of the paper, we focus our attention on the turn-on fluorescence detection of peroxide-based explosives using a redox-active DPP phosphine. Our approach is inspired by recent optical strategies for the analysis of peroxide explosives, involving their conversion to H_2_O_2_ and detection of this target.^28,29^ Notably, oxidation of the triphenylphosphine moiety in (9) is accompanied by a large decrease in its computed HOMO energy (*E*_HOMO_ = −7.26 and −8.47 eV for phosphine and phosphine oxide, respectively), which is sufficient to provide an energetic barrier to intramolecular PET between the phosphine lone pair and DPP core and a large increase in the fluorescence quantum yield (Φ_f_ = 0.20 *versus* 0.88 in acetonitrile for phosphine and phosphine oxide, respectively), corresponding to an ca. sixfold increase in brightness.^34^ Motivated by this redox-mediated fluorescence emission behavior, we envisaged that (9) should therefore be capable of modulating a response to H_2_O_2_ exposure *via* fluorescence enhancement. To test this hypothesis and determine the fluorescence response, we treated (9) with H_2_O_2_ in acetonitrile (Figure 11). Before the reaction with H_2_O_2_ and in light of the ease by which phosphines undergo oxidation, the stability of the probe in solution under aerated conditions was confirmed. The response of 2 μM samples of (9) to increasing concentrations of H_2_O_2_ (0–100 μM) was then evaluated over a 180 min period. Figure 11 illustrates the near-linear emission intensity increment observed with increasing peroxide concentration over the studied timescale. The kinetic profile for oxidation was evaluated for the ≥20 μM H_2_O_2_ experiments, and the corresponding second-order bimolecular rate constant for oxidation, *k*_ox_, was determined to be 0.23(1) M^–1^ s^–1^. From the kinetic data, the response rate of (9) to H_2_O_2_-mediated oxidation is found to be comparable to those of recent molecular probes based on fluorescein.^63−66^ Fluorescence enhancement was readily detected from peroxide concentrations in the ppb range, thus confirming the sensitivity of the method at the required levels for TATP detection and equivalent to state-of-the-art enzyme and deboronation strategies reported previously.^16,28,29^

## Conclusions

The incorporation of amine and phosphine chemistry into DPP molecular architectures has been demonstrated as an effective strategy to enable the fluorescence-based detection of two important and chemically different classes of explosives. For the detection of peroxides, a fluorescence enhancement methodology was developed, inspired by the redox-mediated control of intramolecular PET in a triphenylphosphine-functionalized DPP. After the conversion of peroxide explosives to H_2_O_2_, which is a ubiquitous target metabolite in the detection of this class of explosives, rapid oxidation of a nonemissive DPP triphenylphosphine yielded a highly emissive triphenylphosphine oxide derivative. The kinetic profile for H_2_O_2_ oxidation of the triphenylphosphine was comparable to those of recent molecular probes based on fluorescein, and detection limits in the ppb range confirm the sensitivity of the method at the required levels for TATP analysis, equivalent to state-of-the-art enzyme and deboronation strategies reported previously. For the detection of nitroaromatic explosives, we describe an alternative approach designed to exploit tertiary-amine-mediated donor–accepter charge transfer. Fluorescence quenching from amorphous and seeded films of a triphenylamine DPP upon exposure to NB and DNT vapors was rapid and comparable in sensitivity to our DPP probes reported previously, with upper limits of detection for DNT and NB at 25 °C of 180 ppb and 300 ppm, respectively. Solid-state quenching kinetics for the triphenylamine derivative was analogous to solution, with a faster response from DNT in both amorphous and seeded films. Higher overall quenching at saturation was observed for NB in the amorphous film and for DNT in the seeded environment, leading us to conclude that the selectivity of this material is strongly influenced by the Δ*G* for electron transfer, nitroaromatic vapor pressure, film surface area, and conformational accessibility of emissive sites. Exposure of amorphous and seeded films of a dimethylamine derivative to NB and DNT afforded a remarkable increase in fluorescence emission. To the best of our knowledge, this chemistry represents the first example of a small-molecule probe that facilitates solid-state vapor detection of DNT and NB *via* a fluorescence turn-on mechanism. Enhanced emission in amorphous and seeded films of the dimethylamine derivative was accompanied by changes in absorption and emission spectra and film morphology, which we propose are consistent with the loss of intramolecular charge transfer and formation of a new ground-state charge-transfer complex between DPP and the nitroaromatic. The responses of amorphous and seeded films to NB and DNT vapors and their respective fluorescence enhancement are strongly influenced by the kinetics of complex formation, controlled by the nitroaromatic vapor pressure and reduction potential, film structure, and the accessibility of active fluorophore sites. We are currently seeking to optimize film fabrication and pretreatment to maximize the sensitivity and selectivity of this novel approach, which we anticipate will be of great relevance to those engaged in homeland security and the development of new methods and materials for the optical detection of explosives.

## Supporting Information Available

Photophysical and cyclic voltammetry data for (1)–(8); details of the Stern–Volmer analysis for oxidative quenching of (1)–(8); and optimized geometries of (6)–(8) (PDF)
