# Supplementary material for: Detection of Nitroaromatic and Peroxide-Based Explosives with Amine- and Phosphine-Functionalized Diketopyrrolopyrroles
Source: ACS Appl Mater Interfaces. 2023 May 31;15(23):27915–27. doi: 10.1021/acsami.3c02714 (PMC10273178; doi:10.1021/acsami.3c02714)
Supplement: Supplementary file 1 — am3c02714_si_001.pdf [file am3c02714_si_001.pdf]

Supplementary Information

Detection of nitroaromatic and peroxide based explosives with amine and phosphine functionalised diketopyrrolopyrroles

Monika Warzecha[a], Graeme Morris[b], Andrew J. McLean[b], Jesus Calvo-Castro[c] and Callum J. McHugh*[b]*

[a] *EPSRC CMAC Future Manufacturing Research Hub, c/o Strathclyde Institute of Pharmacy and Biomedical Sciences, Technology and Innovation Centre, 99 George Street, Glasgow, G1 1RD, UK.*

[b] *School of Computing, Engineering and Physical Sciences, University of the West of Scotland, Paisley, PA1 2BE, UK. E-mail: callum.mchugh@uws.ac.uk*

[c] *School of Life and Medical Sciences, University of Hertfordshire, Hatfield, AL10 9AB, UK. E-mail: j.calvo-castro@herts.ac.uk*

Page

S1. Solution-state absorbance and emission spectra of (1-5) 1

S2. Oxidation and reduction CV data for (6-8) 2

S3. Stern-Volmer analysis for oxidative quenching of (1-8) 3

S4. Solid-state Kubelka-Munk absorbance spectra of (1-8) 4

S5. Optimised geometries of (6-8) 5

S1. Solution-state absorbance and emission spectra of (1-5)

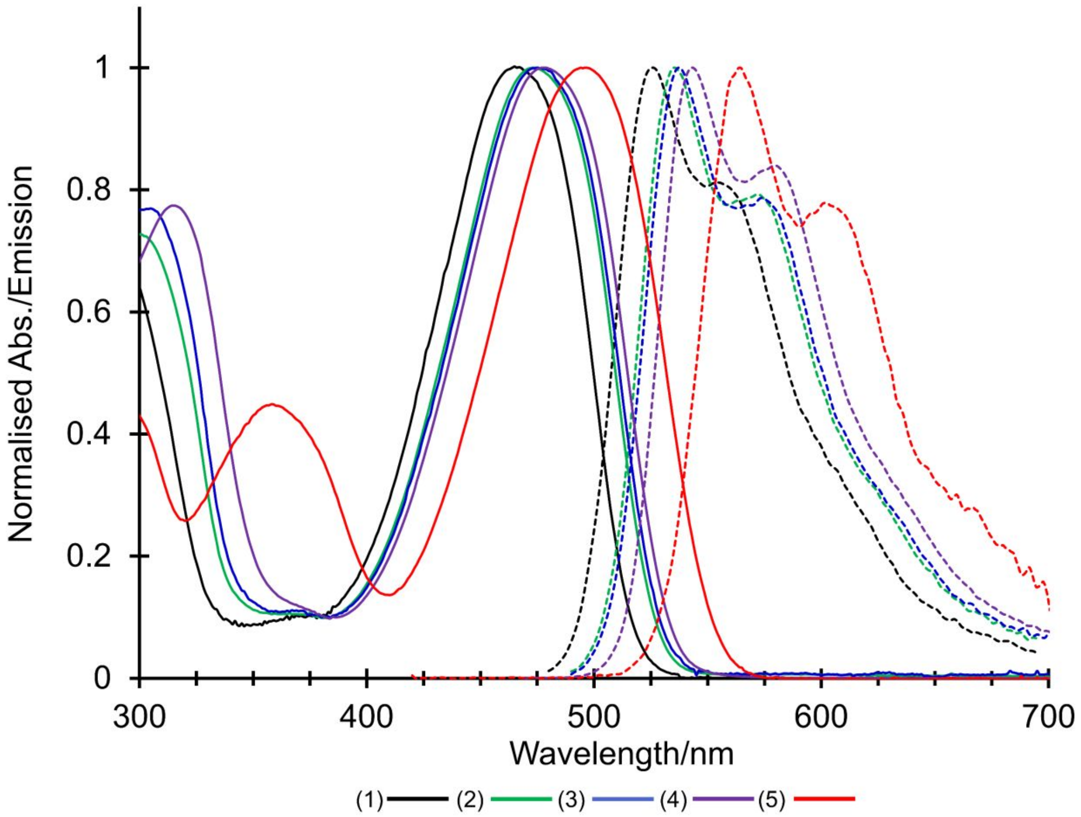

Figure S1 Steady state absorbance (solid lines) and fluorescence emission (dashed lines) spectra of (1-5) in dichloromethane (λ_{exc} = 450, 470, 470, 470 and 490 nm respectively).

S2. Oxidation and reduction CV data for (6-8)

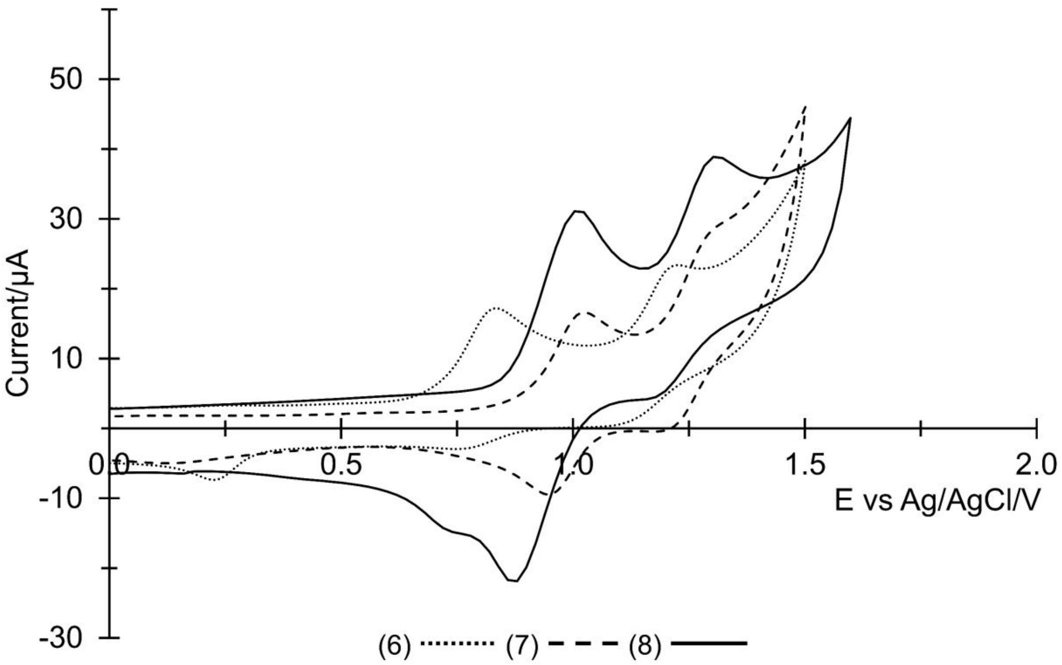

Figure S2 Oxidation and reduction CVs for amines (6-8) in dichloromethane at 100 mVs^{-1} scan rate.

S3. Stern-Volmer analysis for oxidative quenching of (1-8)

Table S1 Stern-Volmer data for oxidative quenching of DPPs (1-8) with nitroaromatics in dichloromethane solution

DPP	Nitroaromatic	k_{sv}/ M^{-1}	k_q/ x 10^9 $M^{-1}s^{-1}$	E (D^+/D) vs. SCE/ eV	E (A/A^-) vs. SCE/ eV	ΔG/ eV
1	NB	25.03	3.92	1.244	-1.116	-0.103
2		22.96	3.86	1.295		-0.006
3		22.72	4.09	1.295		0.003
4		19.22	3.47	1.285		0.005
5		13.31	3.33	1.125		-0.070
6		37.23	8.22	0.836		-0.296
7		5.84	1.55	1.033		-0.162
8		3.79	1.10	0.989		-0.102
1	DNT	49.93	7.81	1.244	-0.901	-0.318
2		39.57	6.65	1.295		-0.221
3		37.06	6.68	1.295		-0.212
4		32.44	5.86	1.285		-0.210
5		38.01	9.50	1.125		-0.285
6		57.92	12.80	0.836		-0.511
7		25.88	6.88	1.033		-0.377
8		23.20	6.72	0.989		-0.317
1	TNT	81.33	12.70	1.244	-0.657	-0.562
2		68.45	11.50	1.295		-0.465
3		55.78	10.10	1.295		-0.456
4		55.91	10.10	1.285		-0.454
5		46.62	11.70	1.125		-0.529
6		74.05	16.30	0.836		-0.755
7		44.50	11.80	1.033		-0.621
8		39.50	11.50	0.989		-0.561

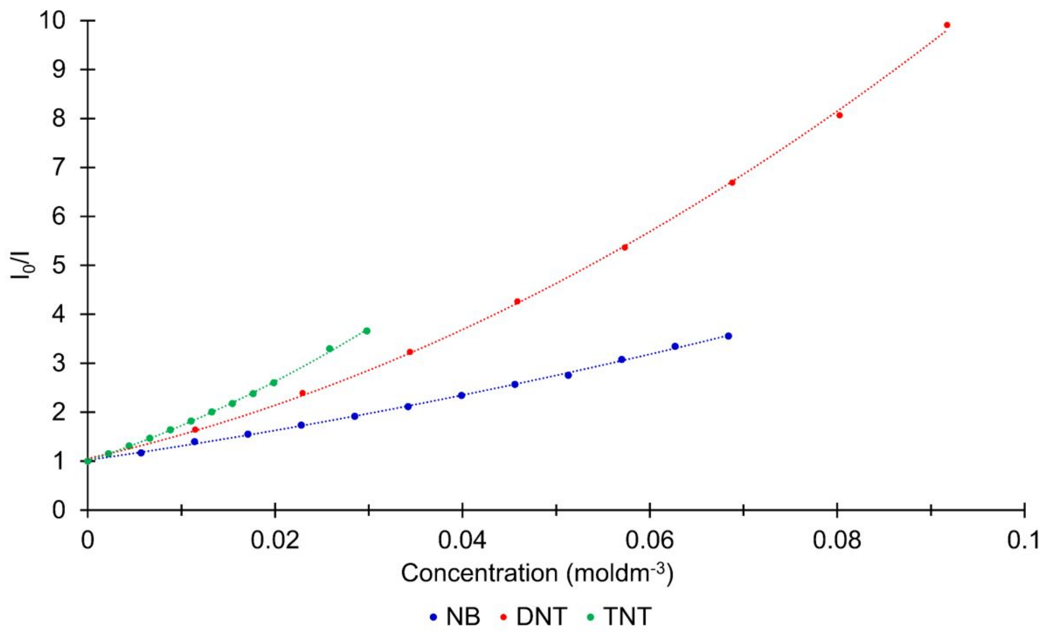

Figure S3 Stern-Volmer plots for oxidative quenching of (6) with high concentrations of NB, DNT and TNT (R^2 > 0.999 in all cases).

S4. Solid-state Kubelka-Munk absorbance spectra of (1-8)

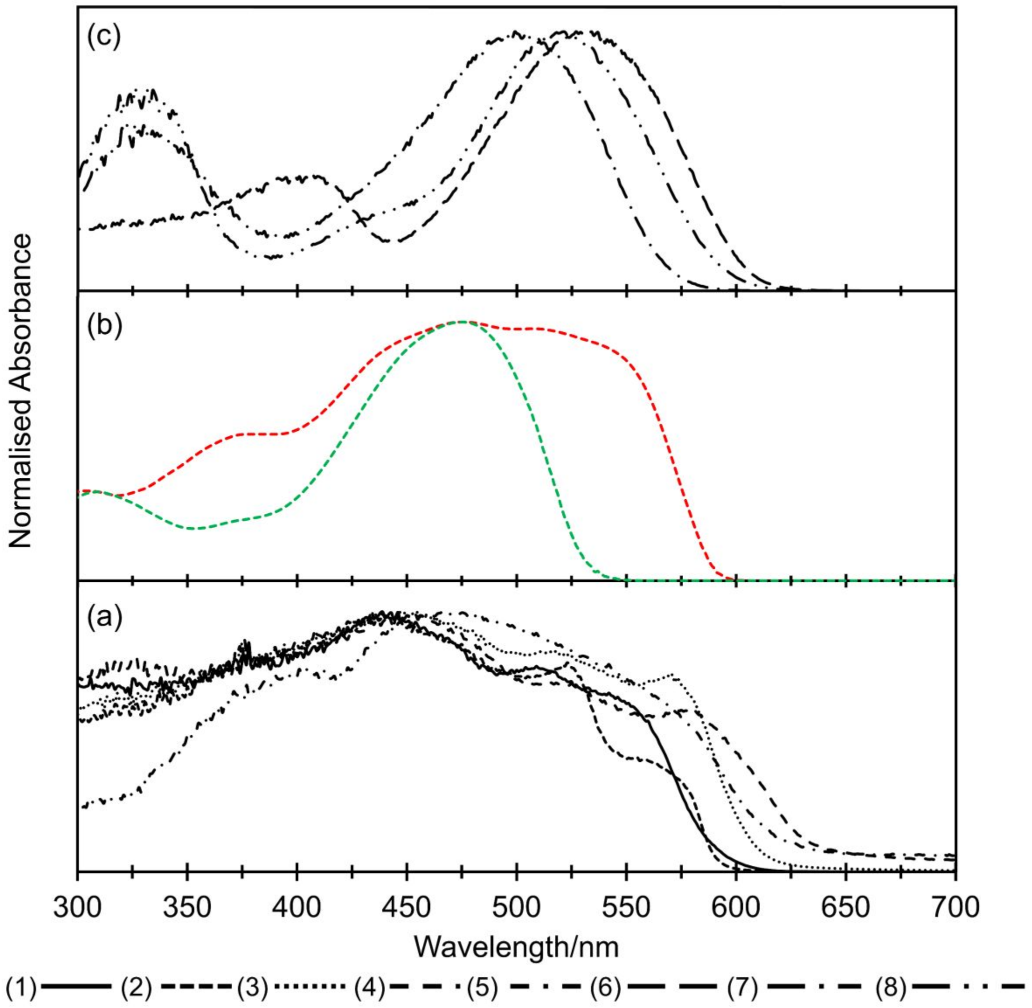

Figure S4 Solid-state Kubelka-Munk absorbance spectra of (a) DPPs (1-5), (b) the α-phase and β-phase (green and red lines respectively) of DPP (2), and (c) DPPs (6-8).

S5. Optimised geometries of (6-8)

Table S2 Neutral geometry of (6), optimised at wB97X-D/6-31G(d) with absolute energy of -1627.282962 hartrees.

Atom	x / Å	y / Å	z / Å
C	-1.253802	-1.06714	1.066619
N	-1.341946	-0.592704	2.410571
C	-0.201703	0.135784	2.760694
C	0.642416	0.138731	1.690375
C	0.061328	-0.632113	0.634898
C	0.995239	-0.818835	-0.346954
C	1.989231	0.510494	1.288517
N	2.16251	-0.147343	0.033652
O	2.821388	1.249582	1.783344
O	-2.168316	-1.636182	0.493558
C	3.197336	0.340804	-0.875748
H	3.683166	-0.494204	-1.381398
H	3.93836	0.813515	-0.226654
C	-2.657944	-0.459386	3.030143
H	-2.615268	-0.765716	4.076461
H	-3.29083	-1.173171	2.497476
C	0.883588	-1.61119	-1.564158
C	0.584228	-3.152639	-3.931009
C	1.936762	-2.388881	-2.059361
C	-0.337051	-1.655935	-2.248719
C	-0.486721	-2.400175	-3.403947
C	1.798391	-3.144436	-3.209811
H	2.874068	-2.442601	-1.514602

**H**	-1.185341	-1.103889	-1.858679
**H**	-1.452456	-2.398608	-3.893543
**H**	2.63749	-3.747106	-3.535105
**C**	0.009538	0.709349	4.09274
**C**	0.437372	1.844505	6.60937
**C**	-0.341242	0.00966	5.25353
**C**	0.606778	1.968713	4.206248
**C**	0.81362	2.532837	5.459344
**C**	-0.130093	0.576428	6.504194
**H**	-0.750385	-0.992817	5.177373
**H**	0.909147	2.49647	3.308543
**H**	1.271746	3.513882	5.536774
**H**	-0.399538	0.023075	7.39875
**C**	-3.208194	0.944809	2.905853
**C**	-4.151371	3.561856	2.628027
**C**	-3.66503	1.403437	1.667648
**C**	-3.227596	1.807671	4.000781
**C**	-3.698292	3.11109	3.863517
**C**	-4.13405	2.704746	1.529198
**H**	-3.648846	0.730803	0.813686
**H**	-2.860791	1.462213	4.964185
**H**	-3.705512	3.774974	4.72303
**H**	-4.489295	3.051054	0.562983
**H**	-4.517873	4.578542	2.519713
**C**	2.653382	1.334246	-1.879332
**C**	1.567825	3.170397	-3.687512
**C**	2.321778	2.626006	-1.462306

**C**	2.435783	0.97164	-3.207743
**C**	1.896955	1.886145	-4.10917
**C**	1.781565	3.538914	-2.360809
**H**	2.490858	2.908356	-0.42617
**H**	2.676506	-0.035876	-3.537245
**H**	1.729692	1.591155	-5.140957
**H**	1.529022	4.540951	-2.026512
**H**	1.146316	3.883551	-4.389953
**H**	0.598145	2.28846	7.587199
**N**	0.448469	-3.874483	-5.096634
**C**	1.507516	-4.772413	-5.506399
**H**	1.690235	-5.570863	-4.771729
**H**	2.446122	-4.228356	-5.663294
**H**	1.236516	-5.237515	-6.45528
**C**	-0.861908	-4.018443	-5.696625
**H**	-0.777128	-4.620594	-6.602431
**H**	-1.270642	-3.043374	-5.985407
**H**	-1.581228	-4.507859	-5.023797

Table S3 Neutral geometry of (7), optimised at wB97X-D/6-31G(d) with absolute energy of -2241.614336 hartrees.

Atom	x / Å	y / Å	z / Å
**C**	0.006944	0.135435	4.051409
**N**	0.111297	0.60563	5.396398
**C**	0.764492	1.839234	5.444547
**C**	1.117433	2.184789	4.173249
**C**	0.706216	1.148346	3.278468

**C**	1.203384	1.412058	2.035596
**C**	1.862458	3.172008	3.408936
**N**	1.915031	2.612748	2.095804
**O**	2.314541	4.266374	3.694113
**O**	-0.608444	-0.866699	3.733937
**C**	2.184733	3.496783	0.962702
**H**	2.776746	2.972152	0.212072
**H**	2.79821	4.302166	1.372757
**C**	-0.838863	0.109149	6.391346
**H**	-0.358393	0.056832	7.368707
**H**	-1.076594	-0.909043	6.076094
**C**	1.088825	0.60602	0.819756
**C**	0.792347	-0.932498	-1.521328
**C**	2.167393	0.422831	-0.053309
**C**	-0.12766	-0.020527	0.53121
**C**	-0.271519	-0.770414	-0.626288
**C**	2.019232	-0.334265	-1.205317
**H**	3.13816	0.842795	0.190294
**H**	-0.961432	0.092618	1.21552
**H**	-1.234245	-1.218789	-0.852327
**H**	2.874815	-0.491197	-1.85567
**C**	1.038668	2.557658	6.691801
**C**	1.546144	3.978037	9.041273
**C**	1.455355	1.886216	7.847519
**C**	0.910611	3.950351	6.714829
**C**	1.159422	4.653691	7.886922
**C**	1.704969	2.59419	9.016208

**H**	1.616249	0.812725	7.820344
**H**	0.62196	4.473337	5.809827
**H**	1.052696	5.734022	7.897316
**H**	2.032739	2.065885	9.906294
**C**	-2.089232	0.959922	6.453279
**C**	-4.362838	2.586527	6.485443
**C**	-3.072561	0.819592	5.470532
**C**	-2.255166	1.920639	7.450984
**C**	-3.387739	2.730558	7.467684
**C**	-4.203073	1.628653	5.486566
**H**	-2.940823	0.073874	4.690975
**H**	-1.490101	2.043055	8.213833
**H**	-3.505995	3.476099	8.248584
**H**	-4.962636	1.509843	4.719357
**H**	-5.24632	3.218214	6.497972
**C**	0.911679	4.045462	0.354966
**C**	-1.490899	5.016957	-0.69374
**C**	0.209852	5.056974	1.015727
**C**	0.399919	3.526568	-0.834214
**C**	-0.796351	4.010617	-1.357256
**C**	-0.9851	5.539699	0.494792
**H**	0.607896	5.460691	1.943086
**H**	0.933497	2.731833	-1.349477
**H**	-1.186095	3.59719	-2.282813
**H**	-1.521731	6.327565	1.015198
**H**	-2.424459	5.394264	-1.100828
**H**	1.738174	4.529856	9.956392

**C**	0.630006	-1.721829	-2.764573
**C**	0.311895	-3.216245	-5.138538
**C**	1.207815	-1.300384	-3.967049
**C**	-0.109423	-2.909483	-2.776594
**C**	-0.275664	-3.64277	-3.94217
**C**	1.063165	-2.035417	-5.134296
**H**	1.774336	-0.373535	-3.99418
**H**	-0.554921	-3.274172	-1.855333
**H**	-0.856923	-4.559033	-3.928843
**H**	1.52249	-1.690036	-6.054762
**N**	0.150493	-3.96097	-6.326184
**C**	-0.032485	-3.297005	-7.564157
**C**	-0.395418	-1.991871	-10.014752
**C**	-0.86393	-2.175785	-7.65149
**C**	0.613622	-3.760253	-8.71468
**C**	0.422418	-3.115742	-9.931088
**C**	-1.031921	-1.524665	-8.867845
**H**	-1.3729	-1.81801	-6.761964
**H**	1.260378	-4.629384	-8.648838
**H**	0.928654	-3.488675	-10.816669
**H**	-1.678339	-0.653558	-8.919746
**H**	-0.536214	-1.485934	-10.964762
**C**	0.140736	-5.377285	-6.282237
**C**	0.122796	-8.176034	-6.199244
**C**	1.06935	-6.067127	-5.496431
**C**	-0.795593	-6.100602	-7.02757
**C**	-0.79406	-7.490055	-6.991285

**C**	1.049473	-7.456026	-5.449418
**H**	1.801424	-5.508362	-4.921762
**H**	-1.519341	-5.567346	-7.63593
**H**	-1.526172	-8.038533	-7.576747
**H**	1.774848	-7.978107	-4.832308
**H**	0.115588	-9.261065	-6.166608

Table S4 Neutral geometry of (8), optimised at wB97X-D/6-31G(d) with absolute energy of -2989.878274 hartrees.

Atom	x / Å	y / Å	z / Å
**C**	-0.496848	1.738034	0.173449
**N**	0.91543	1.925831	0.269509
**C**	1.585477	0.699798	0.239536
**C**	0.652357	-0.292239	0.14717
**C**	-0.651793	0.292998	0.147293
**C**	-1.584864	-0.69913	0.238807
**C**	0.49748	-1.73726	0.172308
**N**	-0.914766	-1.925172	0.268125
**O**	1.293419	-2.654012	0.069213
**O**	-1.292665	2.654928	0.071046
**C**	-1.480466	-3.213486	-0.130029
**H**	-2.316986	-3.473729	0.519363
**H**	-0.681475	-3.937379	0.045892
**C**	1.480863	3.214493	-0.128051
**H**	2.318131	3.473912	0.520665
**H**	0.682111	3.938254	0.049379
**C**	-3.038122	-0.570433	0.348496

**C**	-5.834472	-0.271793	0.513211
**C**	-3.793754	-1.389528	1.196138
**C**	-3.691492	0.424328	-0.386677
**C**	-5.068786	0.56492	-0.306324
**C**	-5.170281	-1.243555	1.272415
**H**	-3.298274	-2.117014	1.831134
**H**	-3.112608	1.084511	-1.022952
**H**	-5.562519	1.32281	-0.907334
**H**	-5.738433	-1.868544	1.955313
**C**	3.038653	0.570705	0.349469
**C**	5.834901	0.271334	0.514536
**C**	3.691711	-0.424963	-0.384751
**C**	3.79452	1.390308	1.196454
**C**	5.170984	1.243929	1.27294
**C**	5.068976	-0.565859	-0.30427
**H**	3.112572	-1.085783	-1.02015
**H**	3.299272	2.118463	1.830871
**H**	5.739294	1.869223	1.955428
**H**	5.562497	-1.324419	-0.904605
**C**	1.905072	3.227685	-1.580792
**C**	2.652085	3.171587	-4.274003
**C**	0.935236	3.294935	-2.584528
**C**	3.249603	3.131686	-1.938127
**C**	3.62261	3.104559	-3.279477
**C**	1.306608	3.266407	-3.923857
**H**	-0.112802	3.368889	-2.30581
**H**	4.009376	3.06522	-1.163529

**H**	4.672655	3.027466	-3.545666
**H**	0.545446	3.322235	-4.696469
**H**	2.942073	3.150236	-5.3204
**C**	-1.906173	-3.22535	-1.582367
**C**	-2.655823	-3.166815	-4.274828
**C**	-0.937095	-3.288166	-2.587119
**C**	-3.251272	-3.132355	-1.93833
**C**	-3.625592	-3.104171	-3.279288
**C**	-1.309743	-3.258311	-3.926047
**H**	0.111403	-3.359747	-2.309492
**H**	-4.010475	-3.068413	-1.162939
**H**	-4.676105	-3.029599	-3.544343
**H**	-0.549094	-3.310356	-4.699419
**H**	-2.946829	-3.14449	-5.320922
**C**	7.307428	0.12585	0.592706
**C**	10.109362	-0.147026	0.756529
**C**	7.905666	-1.136918	0.661411
**C**	8.141792	1.24852	0.605015
**C**	9.520397	1.120027	0.692323
**C**	9.283816	-1.276109	0.734886
**H**	7.281517	-2.026157	0.669398
**H**	7.705528	2.241352	0.536687
**H**	10.150672	2.003506	0.703143
**H**	9.729638	-2.264075	0.789042
**C**	-7.307037	-0.126622	0.591385
**C**	-10.10894	0.145766	0.755738
**C**	-8.14115	-1.249472	0.604381

**C**	-7.905496	1.136056	0.659581
**C**	-9.283662	1.275018	0.733326
**C**	-9.519765	-1.121204	0.691921
**H**	-7.704674	-2.242238	0.536441
**H**	-7.281481	2.025395	0.666972
**H**	-9.72969	2.262912	0.78715
**H**	-10.149914	-2.004768	0.703269
**N**	-11.512318	0.285117	0.845832
**N**	11.512729	-0.286647	0.84619
**C**	12.067801	-1.241273	1.732898
**C**	13.15601	-3.132118	3.488957
**C**	13.167872	-2.013885	1.346695
**C**	11.518718	-1.423027	3.006378
**C**	12.054866	-2.370229	3.870382
**C**	13.710863	-2.943483	2.225794
**H**	13.594099	-1.87882	0.3576
**H**	10.668283	-0.821366	3.311187
**H**	11.615263	-2.502009	4.854716
**H**	14.566367	-3.534951	1.912964
**H**	13.577477	-3.86557	4.169171
**C**	12.357534	0.54213	0.070105
**C**	14.023795	2.184729	-1.471145
**C**	12.028201	0.840027	-1.256552
**C**	13.529931	1.073649	0.617658
**C**	14.358457	1.879258	-0.15453
**C**	12.851826	1.664186	-2.013783
**H**	11.122655	0.424416	-1.687216

**H**	13.787628	0.84898	1.647766
**H**	15.2666	2.282481	0.284022
**H**	12.580757	1.887871	-3.041423
**H**	14.669476	2.821119	-2.068107
**C**	-12.357196	-0.543838	0.070039
**C**	-14.023341	-2.186703	-1.470852
**C**	-12.02788	-0.842056	-1.256598
**C**	-13.529537	-1.075192	0.617786
**C**	-14.357992	-1.880935	-0.154268
**C**	-12.851421	-1.666362	-2.013727
**H**	-11.122302	-0.426492	-1.687253
**H**	-13.787218	-0.850313	1.647846
**H**	-15.266147	-2.284108	0.284312
**H**	-12.579444	-1.891116	-3.044781
**H**	-14.669107	-2.823245	-2.067568
**C**	-12.067231	1.239838	1.732541
**C**	-13.154995	3.130909	3.488616
**C**	-11.517701	1.421897	3.00579
**C**	-13.167528	2.012247	1.346579
**C**	-13.710292	2.941965	2.225698
**C**	-12.053636	2.369209	3.869798
**H**	-10.667094	0.820379	3.310407
**H**	-13.594097	1.876936	0.357666
**H**	-14.565971	3.533282	1.913064
**H**	-11.613686	2.501231	4.853944
**H**	-13.576289	3.864456	4.168835
